# *Trametes versicolor* Protein YZP Activates Regulatory B Lymphocytes – Gene Identification through De Novo Assembly and Function Analysis in a Murine Acute Colitis Model

**DOI:** 10.1371/journal.pone.0072422

**Published:** 2013-09-03

**Authors:** Yen-Chou Kuan, Ying-Jou Wu, Chih-Liang Hung, Fuu Sheu

**Affiliations:** 1 Center for Biotechnology, National Taiwan University, Taipei, Taiwan; 2 Department of Horticulture, National Taiwan University, Taipei, Taiwan; Northwestern University Feinberg School of Medicine, United States of America

## Abstract

**Background:**

*Trametes versicolor* (Yun-Zhi) is a medicinal fungus used as a chemotherapy co-treatment to enhance anti-tumor immunity. Although the efficacies of *T. versicolor* extracts have been documented, the active ingredients and mechanisms underlying the actions of these extracts remain uncharacterized.

**Results:**

We purified a new protein, YZP, from the fruiting bodies of *T. versicolor* and identified the gene encoding YZP using RNA-seq and de novo assembly technologies. YZP is a 12-kDa non-glycosylated protein comprising 139 amino acids, including an 18-amino acids signal peptide. YZP induced a greater than 60-fold increase in IL-10 secretion in mice B lymphocytes; moreover, YZP specifically triggered the differentiation of CD1d^+^ B cells into IL-10-producing regulatory B cells (Bregs) and enhanced the expression of CD1d. YZP-induced B cells suppressed approximately 40% of the LPS-activated macrophage production of inflammatory cytokines in a mixed leukocyte reaction and significantly alleviated the disease activity and colonic inflammation in a DSS-induced acute colitis murine model. Furthermore, YZP activated Breg function via interaction with TLR2 and TLR4 and up-regulation of the TLR-mediated signaling pathway.

**Conclusions:**

We purified a novel Breg-stimulating protein, YZP, from *T. versicolor* and developed an advanced approach combining RNA-seq and de novo assembly technologies.to clone its gene. We demonstrated that YZP activated CD1d^+^ Breg differentiation through TLR2/4-mediated signaling pathway, and the YZP-stimulated B cells exhibited anti-inflammatory efficacies in vitro and in murine acute colitis models.

## Introduction

*Trametes versicolor*, formerly known as *Coriolus versicolor*, is a medicinal fungus widely used as a co-treatment to enhance immunity in cancer patients [1]. This fungus, known as “Yun-Zhi” in China, “kawaratake” in Japan, and “turkey tail mushroom” in western countries, has been used as a therapeutic agent worldwide. The immune-stimulating and anti-tumor effects of *T. versicolor* extracts have been extensively researched in in vitro, in vivo and clinical studies [1]–[3]. *T. versicolor* extracts activate the effector functions of T lymphocytes [4], B lymphocytes [5], monocytes [6], and natural killer cells [7]. Moreover, *T. versicolor* extracts suppress cancer cell growth and increase the tumor-killing activities of immune cells [8]–[12]. Polysaccharopeptides have been identified as the primary active ingredients in these extracts; however, these compounds only comprised mixtures of β-glucan with approximately 30% (w/w) protein [13]. We have previously demonstrated that, in addition to polysaccharides, the proteins from medicinal fungi and plants also possess remarkable pharmacological activities [14]–[17]. Therefore, it is of great interest to identify proteins with therapeutic value in *T. versicolor*.

Next-generation sequencing (NGS) is an emerging technology that has broadened the scope of genomic research in recent years [18]. Current NGS systems, such as Solexa/Illumina of Illumina, 454 of Roche, and SOLiD of ABI, are capable of sequencing millions of DNA fragments and processing more than 10^9^ bases of data within days, improving efficiency and data throughput [19]. NGS has been used in a variety of research studies, such as de novo transcriptome analysis, genome re-sequencing, and microRNA analysis [20]–[24]. De novo transcriptome assembly is a technique used to generate the entire transcriptome of an organism through RNA sequencing (RNA-seq). Briefly, mRNA is purified from an organism, reverse transcribed to establish a cDNA library, fragmented into short reads, and sequenced on NGS platforms. The full-length sequences (contigs) can be obtained from assembling the short reads, and the contigs can subsequently be annotated and further analyzed [25].

Antibody (Ab)-secreting B lymphocytes act as effector cells in humoral immunity as well as antigen-presenting cells (APCs) that modulate T-cell responses through co-stimulation and cytokine production [26]. During the last decade, Interleukin (IL)-10-producing B cells that possess immune-suppressive functions have been identified in both human autoimmune diseases and murine models of those diseases [27], [28]. This distinct B lymphocyte subset is referred to as regulatory B cells (Bregs). Although the ontogeny and development of Bregs remain undefined, it is known that Breg differentiation is induced through toll-like receptors (TLRs) and/or co-stimulatory signals depending on the responding B-cell subsets [29], [30].

In this study, we purified a **Y**un-**Z**hi **p**rotein (**YZP**) via chromatography and cloned its gene using a novel approach combining N-terminal amino acid analysis, de novo assembly, and PCR cloning techniques. We demonstrated that YZP promotes the differentiation of IL-10-producing Bregs and confirmed the regulatory function of YZP-induced B lymphocytes using a DSS-induced acute colitis murine model. Furthermore, we elucidated the interaction between YZP and TLRs in TLR2 and TLR4 gene knockout mice and using fluorescein isothiocyanate (FITC)-labeled YZP.

## Results

### Purification of YZP from *T. versicolor* fruiting bodies

**Figure 1:**
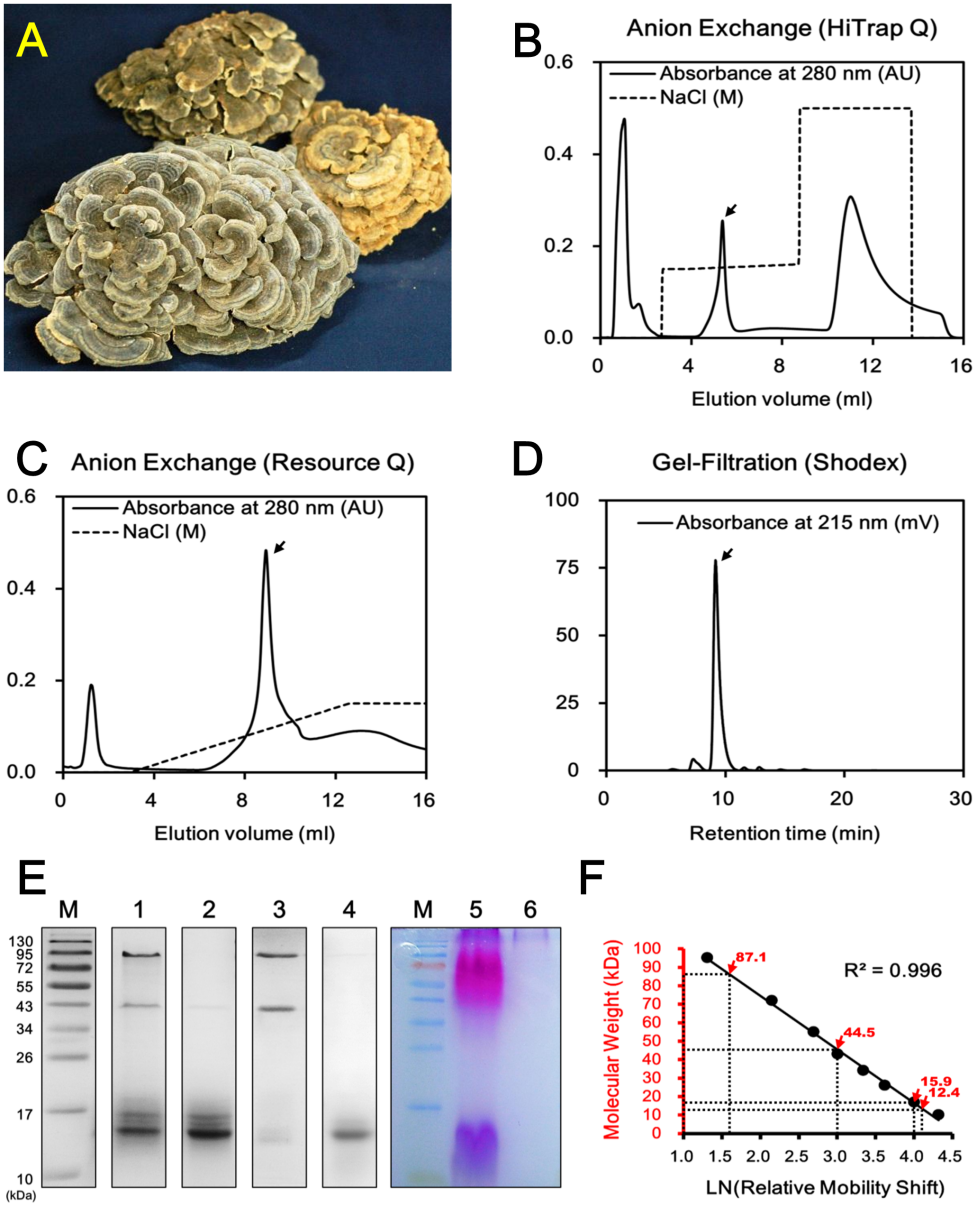
Purification and biochemical characterization of YZP. **A.** Fruiting bodies of *Trametes versicolor* (Yun-Zhi). **B.** Chromatogram of the crude protein extract of *T. versicolor* fractionated on a HiTrap Q anion exchange column. **C.** Chromatogram of the pooled YZP-containing fractions fractionated on a Resource Q anion exchange column. **D.** Chromatogram of the purified YZP samples analyzed by PROTEIN KW-802.5 SHODEX gel-filtration chromatography. **E.** SDS-PAGE analysis of the crude protein extracts from *T. versicolor* (lanes 1 and 5), the YZP-containing fraction (lane 2), non-YZP fraction (lane 3), and purified YZP (lanes 4 and 6). Lanes 1 to 4 were stained with CBR, whereas lanes 5 and 6 were stained with PAS reagent. Lane M was loaded with PageRuler Prestained Protein Ladder #SM0671. **F.** The calibration curve was established via linear regression of the molecular weights of the proteins present in the PageRuler Prestained Protein Ladder #SM0671 versus the nature logarithm of their relative mobility shifts. The molecular weights of *T. versicolor* proteins are indicated with red arrows.

The crude proteins extracted from *T. versicolor* fruiting bodies (**Figure 1A**) were analyzed by SDS-PAGE, and 4 major bands (lane 1 in **Figure 1E**) with molecular weights (MW) of 87, 45, 16, and 12 kDa were detected. To separate these proteins, the crude extracts were fractionated on a HiTrap Q anion exchange column (**Figure 1B**). When eluted with 0.15 to 0.16 M NaCl-Tris buffer (N-TB, pH 8.2), a single peak comprising the 16 and 12 kDa proteins (lane 2 in **Figure 1E**) was obtained, whereas the 87 and 45 kDa proteins were eluted with 0.5 M N-TB (lane 3 in **Figure 1E**). The fractions containing the 16- and 12-kDa proteins were pooled and re-dialyzed into TB for further purification on a Resource Q anion exchange column (**Figure 1C**). The column was eluted with 0.15 to 0.16 M N-TB, and a sample containing the 12-kDa protein was obtained (lane 4 in **Figure 1E**). This sample was analyzed with a Shodex gel-filtration column (**Figure 1D**), and the purity of the sample was greater than 95%, based on the calculation of the peak area relative to the total area under the curve. The immuno-modulating activity of the crude proteins extract, the lower-MW fraction (YZP-enriched), and the higher-MW fraction (non-YZP) was analyzed using murine peritoneal macrophages (MΦ) and splenocytes. The crude proteins extract stimulated TNF-α production in MΦ and increased cellular enzyme activity in splenocytes. These effects were significantly elevated in YZP-enriched fraction purified through HiTrap Q column (Figure S1). The purity of YZP was significantly improved from ∼90% to above 96% through further purification with Resource Q column; however, only mild enhancement in immuno-modulating activity was observed.

**Figure 2:**
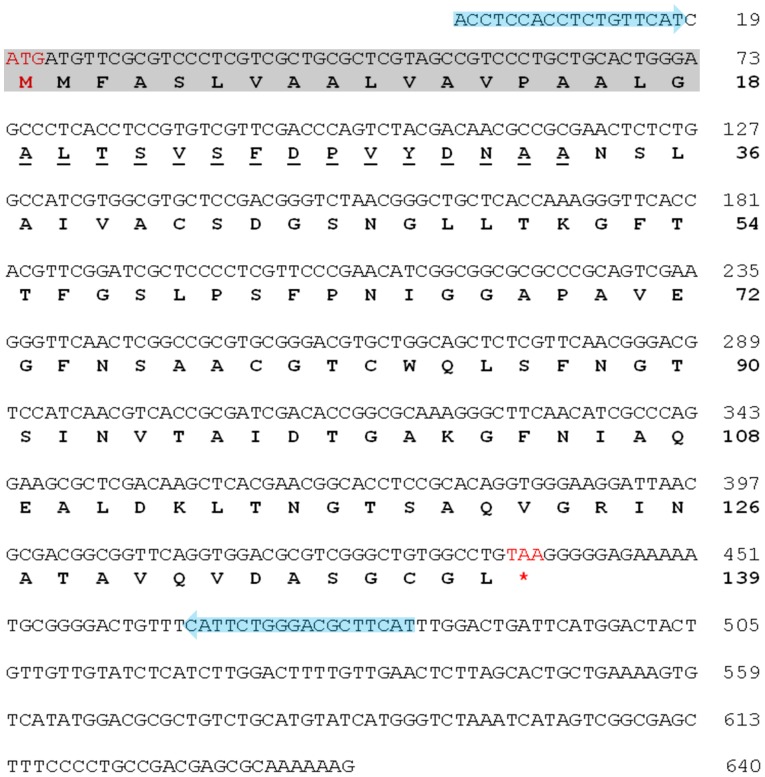
Complete nucleotide and amino acid sequences of YZP. RNA-seq and de novo contig assembly were conducted to identify the YZP gene. The complete nucleotide sequence of the YZP cDNA was confirmed by PCR cloning using the primers shown in Supporting Information Table S1 and Table S2, and is presented in plain capital letters with the start and termination codons presented in red. The nucleotide sequences of one set of cloning primers (YZP1) are indicated with blue arrows. The translated amino acid sequence is presented in bold capital letters, whereas the N-terminal amino acids are underlined. The putative signal peptide predicted using the SignalP 4.0 Server (Supporting Information Figure S3) is indicated with a grey background.

SDS-PAGE followed by periodic acid Schiff staining was performed to confirm the lack of polysaccharide contamination in the protein samples. Purple bands corresponding to positive signals for carbohydrate content were observed for the crude protein (lane 5 in **Figure 1E**). However, under the same conditions, the purple bands at the 12-kDa position diminished in the sample corresponding to the purified 12-kDa protein, suggesting that after purification via 2 chromatographic steps, the 12-kDa protein was free of carbohydrate content (lane 6 in **Figure 1D**). This protein significantly activated IL-10 production and cell proliferation in mice splenic immune cells (Figure S2) and was subsequently referred to as the **Y**un-**Z**hi **p**rotein (**YZP**). Endotoxin contamination in the YZP sample was less than 0.013 EU/mg, as examined with a ToxinSensor™ Chromogenic LAL Endotoxin Assay Kit. The following protein yields were generated from each purification step: 0.21±0.11 g of crude protein was extracted from 1 kg of *T. versicolor* fruiting bodies; 85.3±36.1 mg of low MW proteins fraction was isolated from 1 g of crude proteins; and 0.6±0.1 mg of YZP was purified from 1 mg of low MW proteins. In summary, the yield of purified YZP was 9.8±2.4 mg per kg of *T. versicolor* fruiting bodies. Edman degradation permitted the identification of the first 15 amino acids (aa) of the N-terminus of YZP as [A], [L], [T], [S], [V], [S], [F], [D], [P], [V], [Y], [D], [N], [A], and [A] (underlined letters in **Figure 2**). The N-terminal amino acid sequence was used as the target for YZP gene identification.

### De novo assembly of contigs and gene identification of YZP

Total mRNA purified from fresh *T. versicolor* fruiting bodies was used for RNA-seq on an Illumina GAII platform. A total of 27,157,946 reads with an accumulating length of 2,444,215,140 base pairs (bp) and a GC content of 59.4% were obtained, of which 26,206,475 matched reads were de novo assembled into 38,964 contigs. The average length of the contigs was 639 bp, and the minimum, N50, and maximum lengths were 158, 828, and 6,717 bp, respectively (**Table 1**). The contigs were translated into amino acids sequences to identify a sequence matching the N-terminus of YZP. The nucleotide sequences for each contig had 2 possible orientations and 3 translation start sites. To include all possibilities, each contig was translated into 6 amino acids sequences using 3 forward 5′ to 3′ starting sites (+1, +2 and +3) and 3 reverse 3′ to 5′ starting sites (–1, –2 and –3). One contig (#9142) that encoded the N-terminus of YZP was identified with the +2-starting site and was therefore proposed as the gene encoding YZP. The primer sets were designed based on the nucleotide sequence of contig #9142 (Table S1 and S2) and were used for PCR cloning of the YZP gene. The complete cDNA sequence of the YZP gene was cloned and was determined to be completely identical to the nucleotide sequence of contig #9142 (**Figure 2**). The YZP gene is composed of 640 bp, including a 417-bp open reading frame encoding a 139 amino acids sequence. The first 18 amino acids comprised a signal peptide as predicted by the SignalP 4.0 Server (http://www.cbs.dtu.dk/services/SignalP/); thus, the N-terminus of YZP occurs at the 19th amino acids position. The theoretical MW deduced from the amino acids sequence is 12.1 kDa (Figure S3), consistent with the MW determined from the SDS-PAGE relative mobility shift calculation (**Figure 1F**). The complete cDNA sequence of YZP has been submitted to the GenBank® database under accession number KC297708.

**Table 1:** Statistics of *T. versicolor* RNA-seq and de novo assembly.

Output statistics	Total numbers of	Contig statistics	Length in bp
**Nucleotides**	2,444,215,140	**Minimum**	158
**Adenine & Thymine**	985,018,701	**Average**	639
**Cytosine & Guanine**	1,451,863,793	**Maximum**	6,717
**Reads**	27,157,946	**N75**	443
**Matched reads**	26,206,475	**N50**	828
**Contigs**	38,964	**N25**	1,747

### YZP induced IgM-producing plasma cells and IL-10^+^ Bregs differentiation

**Figure 3:**
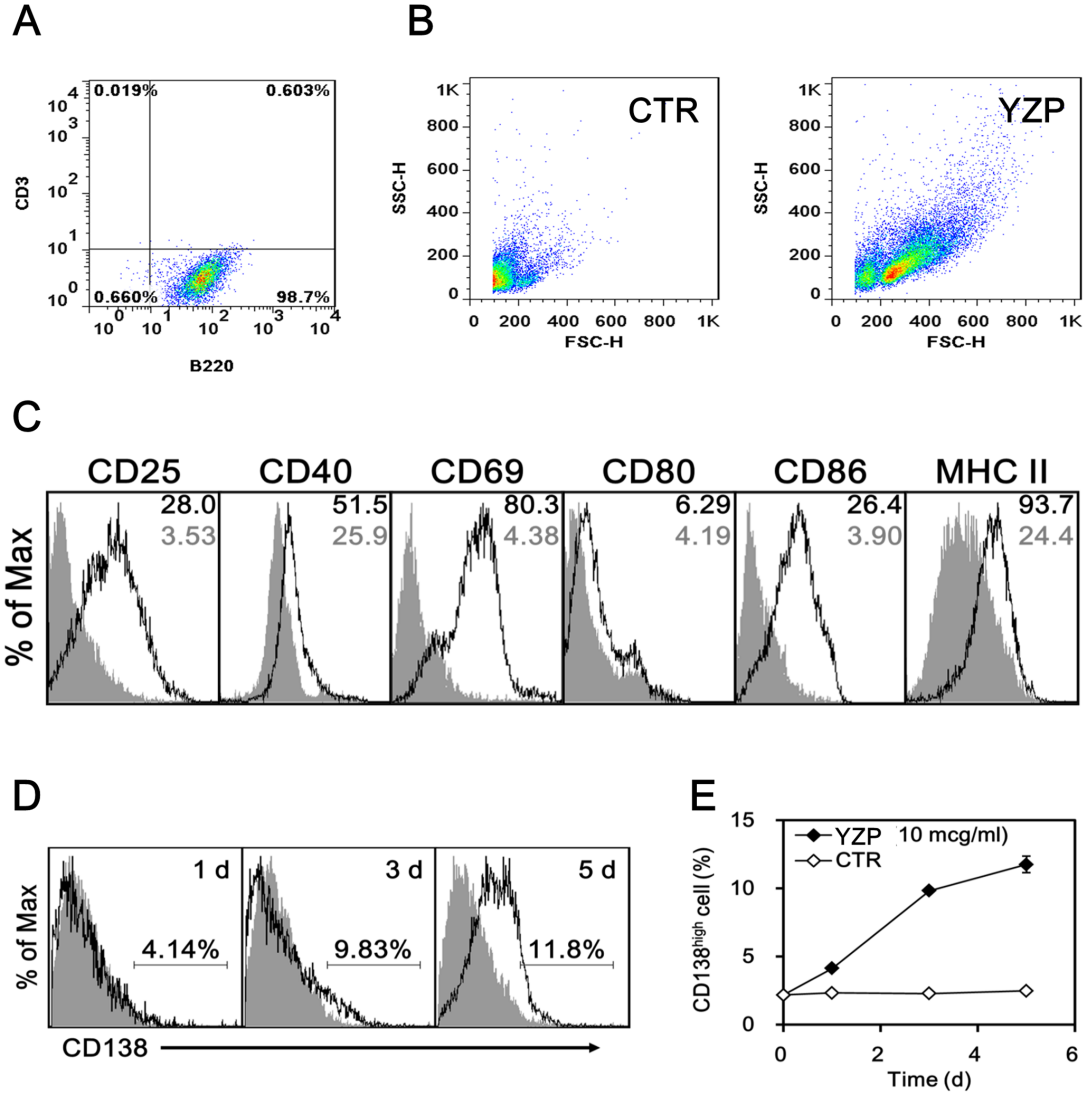
YZP activates plasma cell differentiation and IgM production in mice B cells. **A.** The purity of the MACS-purified CD19^+^ B cells was examined by analyzing the expression of CD3 and B220 via flow cytometry. **B.** The MACS-purified CD19^+^ B cells were treated with YZP (20 μg/mL) for 24 h, and the cell size and granularity was examined by analyzing the forward scatter (FSC) and side scatter (SSC) via flow cytometry. **C.** MACS-purified CD19^+^ B cells were treated with YZP (20 μg/mL) for 24 h, followed by analysis of the expression levels of the indicated surface markers by flow cytometry. The shaded gray color indicates untreated B cells, whereas the solid line indicates B cells treated with YZP. The numbers indicate the geometric mean fluorescence intensity. **D. and E.** MACS-purified CD19^+^ B cells were treated with YZP (20 μg/mL) for the indicated periods, and subsequently the expression level of CD138 was analyzed via flow cytometry. The numbers indicate the percentage of gated CD138^high^ cells.

Our preliminary experiments showed that YZP markedly stimulated murine B lymphocytes (Figure S2); therefore, we examined the effect of YZP on murine B lymphocyte differentiation. Magnetic-activated cell sorting (MACS)-purified CD19^+^ B cells were cultured with YZP (20 μg/mL), and surface marker expression was analyzed. The purity of the B cell cultures used in this study was constantly examined and was always above 98%; a representative result is shown in **Figure 3A**. Drastic increase in cell size (FSC) and granularity (SSC) (**Figure 3B**) as well as significant increase in the expression of the lymphocyte activation markers CD25 and CD69; the APC activation markers CD40, CD80, and CD86; and the major histocompatibility complex class II (MHC II) was observed after 24 h of YZP stimulation (**Figure 3C**). CD138 is known as a marker of mature Ab-secreting plasma cells; therefore, we examined the expression of this marker in a time-course experiment to clarify whether YZP induces B-cell maturation. The population of CD138^high^ B cells increased steadily from 2.2±0.1% to 4.1±0.1% after 1 day and further increased to 9.3±0.1% and 11.8±0.6% after 3 and 5 days of YZP stimulation, respectively (**Figure 3D and 3E**). This finding was consistent to the elevated FSC and SSC since plasma cell formation would enrich the content and complexity of the cell.

**Figure 4:**
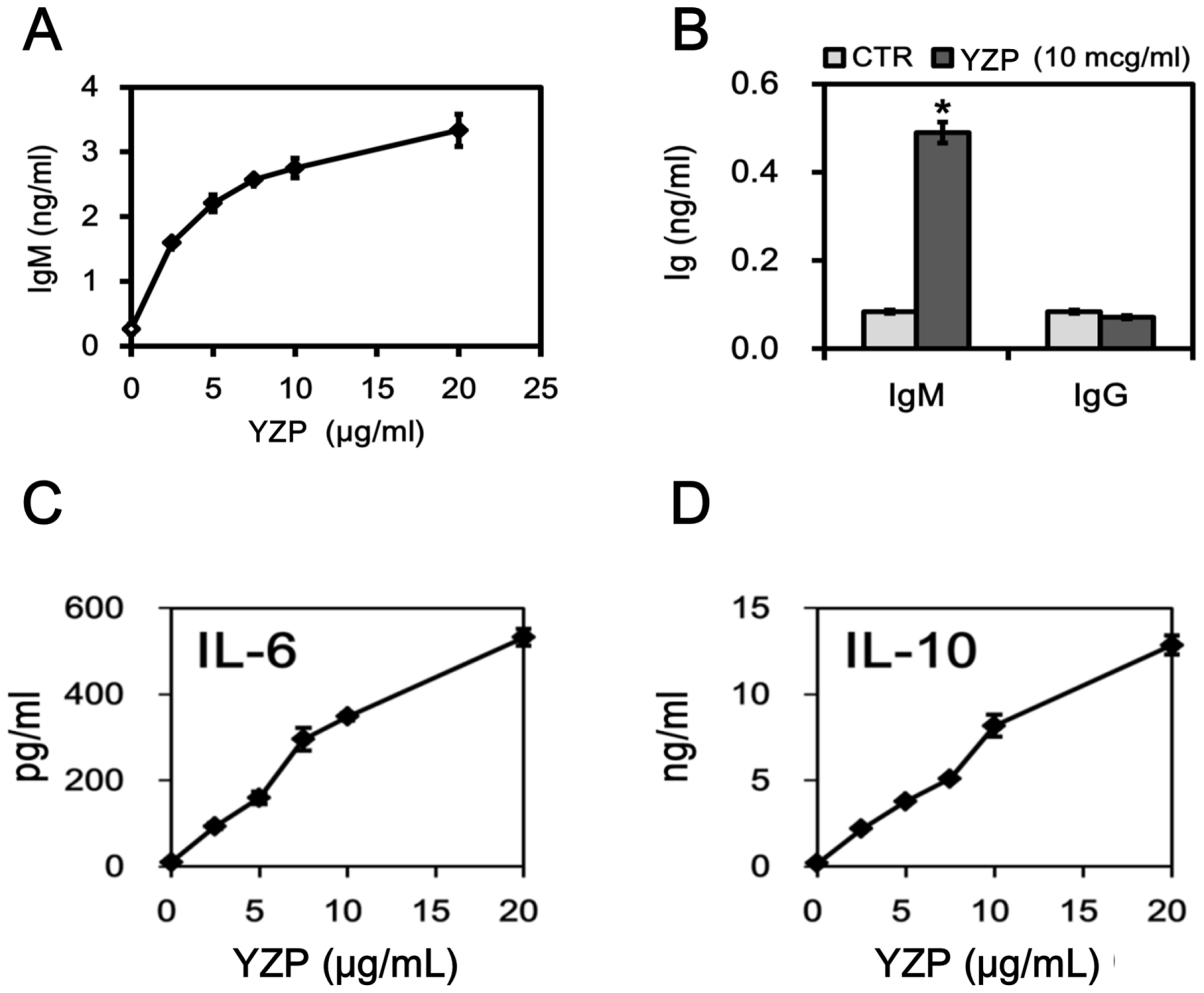
YZP induces IL-6 and IL-10 production in mice B cells. **A.** MACS-purified CD19^+^ B cells were treated with the indicated dosages of YZP for 72 h, and the culture supernatant was then collected for IgM measurements. **B.** MACS-purified CD19^+^ B cells were treated with YZP (10 μg/mL) for 72 h, and subsequently the culture supernatant was collected for IgM and IgG measurements. **C. and D.** MACS-purified CD19^+^ B cells were treated with the indicated dosages of YZP for 72 h, and subsequently the culture supernatant was collected for cytokine measurements.

**Figure 5:**
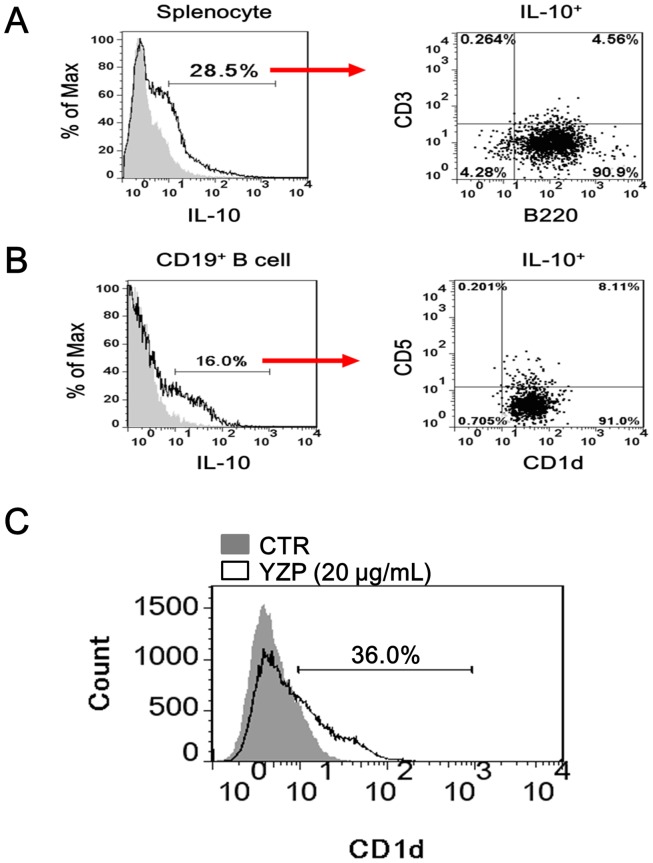
YZP stimulates CD1d^+^ B cells to produce IL-10 and increases CD1d expression in mice B cells. **A.** Mice splenocytes were treated with YZP (20 μg/mL) for 48 h, and subsequently the cells were harvested for PerCP-Cy5.5-labeled anti-mouse CD3 and FITC-labeled anti-mouse B220 surface staining and PE-labeled IL-10 intracellular staining. **B.** MACS-purified CD19^+^ B cells were stimulated with YZP (20 μg/mL) for 48 h, and subsequently the cells were harvested for PerCP-Cy5.5-labeled anti-mouse CD5 and FITC-labeled anti-mouse CD1d surface staining and PE-labeled IL-10 intracellular staining. **C.** The CD1d expression of B cells treated with YZP (20 μg/mL) or untreated for 48 h. The numbers indicate the percentage of gated CD1d^+^ cells.

We examined the immunoglobulin (Ig) production ability of YZP-stimulated B cells and discovered a dose-dependent secretion of IgM (**Figure 4A**), whereas IgG production was unaltered (**Figure 4B**). In addition to Ab-secretion, B lymphocytes also modulate immune responses through the production of cytokines [26]. We screened for the presence of cytokines in the culture supernatant of B cells stimulated with YZP (2.5–20 μg/mL) and observed significant productions of IL-6 (**Figure 4C**) and IL-10 (**Figure 4D**). Intracellular staining experiments also confirm the capability of YZP to activate IL-10 expression by splenocytes and purified B cells (**Figure 5C and 5D**).

IL-10^+^ Breg subsets, such as the CD1d^+^ marginal zone (MZ) B cells and the CD1d^hi^CD5^+^ B10 cells, have been identified in mice [27]. In the present study, we showed that YZP primarily stimulated the CD1d^+^ MZ B cells (>99% of total IL-10^+^ cells) to express IL-10, and approximately 8% of the CD1d^+^ cells were CD5^+^ B10-like cells (**Figure 5B**). In addition, we also showed that YZP enhanced the expression of CD1d on B cells (**Figure 5C**).

### Regulatory function of YZP-induced B lymphocytes

**Figure 6:**
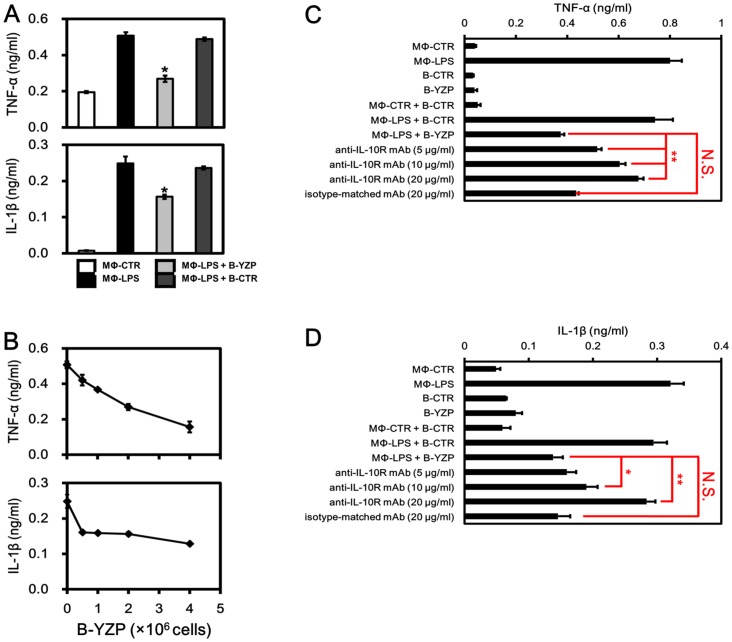
YZP-induced B cells inhibit inflammatory cytokine release from LPS-activated macrophages. **A.** Mice peritoneal macrophages (2×10^6^) were stimulated with or without LPS (1 μg/mL) for 12 h and subsequently co-cultured with or without an equal number (2×10^6^) of YZP-induced B cells or control B cells for 12 h. The culture supernatant was subsequently collected for TNF-α and IL-1β measurements. **B.** Mice peritoneal macrophages (2×10^6^) were stimulated with LPS (1 μg/mL) for 12 h and were subsequently co-cultured with the indicated numbers of YZP-induced B cells for 12 h. The culture supernatant was collected for TNF-α and IL-1β measurements. **C. and D.** Mice peritoneal macrophages (1×10^6^) were stimulated with or without LPS (1 μg/mL) for 12 h and subsequently co-cultured with or without 2×10^6^ YZP-induced B cells or control B cells for 12 h. The culture supernatant was subsequently collected for TNF-α and IL-1β measurements. The data is presented as the mean ± SD, where the asterisks indicate the statistical differences (*, *p*<0.05; **, *p*<0.01; N.S., non-significant) among groups.

YZP-induced B cells (B-YZP) comprised approximately 16% of IL-10^+^ Bregs (**Figure 5B**). To determine if B-YZP has immune suppressive activity, LPS-activated macrophages (MΦ-LPS) were co-cultured with B-YZP or untreated B cells (B-CTR) for 12 h, and the inflammatory cytokines present in the culture supernatant were quantified. Compared with untreated cells (MΦ-CTR), MΦ-LPS produced 2.6- and 33-fold higher amounts of TNF-α and IL-1β, respectively, in macrophage cultures. Co-culturing MΦ-LPS with B-YZP resulted in a significant reduced of TNF-α and IL-1β production by 46.9% and 37.0%, respectively (**Figure 6A**). Moreover, B cell-dependent reductions in TNF-α and IL-1β production were observed when MΦ-LPS were co-cultured with different amounts of B-YZP (**Figure 6B**). Collectively, these results confirmed the regulatory activity of B-YZP.

To test whether the B-YZP-produced IL-10 was related to the cytokine-suppressing effect, we conducted a neutralization experiment in which MΦ-LPS was treated with anti-IL-10R antibodies (5, 10 and 20 μg/mL) or isotype-matched control antibodies (20 μg/mL) prior to the mix leukocyte reaction. B-YZP reduced TNF-α and IL-1β production as expected. However, when the IL-10R presented on MΦ-LPS were blocked, the cytokine-suppressing effect of B-YZP was significantly inhibited (**Figure 6C and 6D**). This finding clearly demonstrated the indispensable role of IL-10 in the regulatory effect of B-YZP.

The in vivo effect of B-YZP was examined in an animal model of DSS-induced colitis. C57BL/6 mice were intraperitoneally injected with PBS, B-YZP, or B-CTR at 24 h prior to the ingestion of 3% DSS. The body weight and disease activities were recorded daily for 7 days, and the mice were subsequently euthanized to assess the resulting colonic damage.

**Figure 7:**
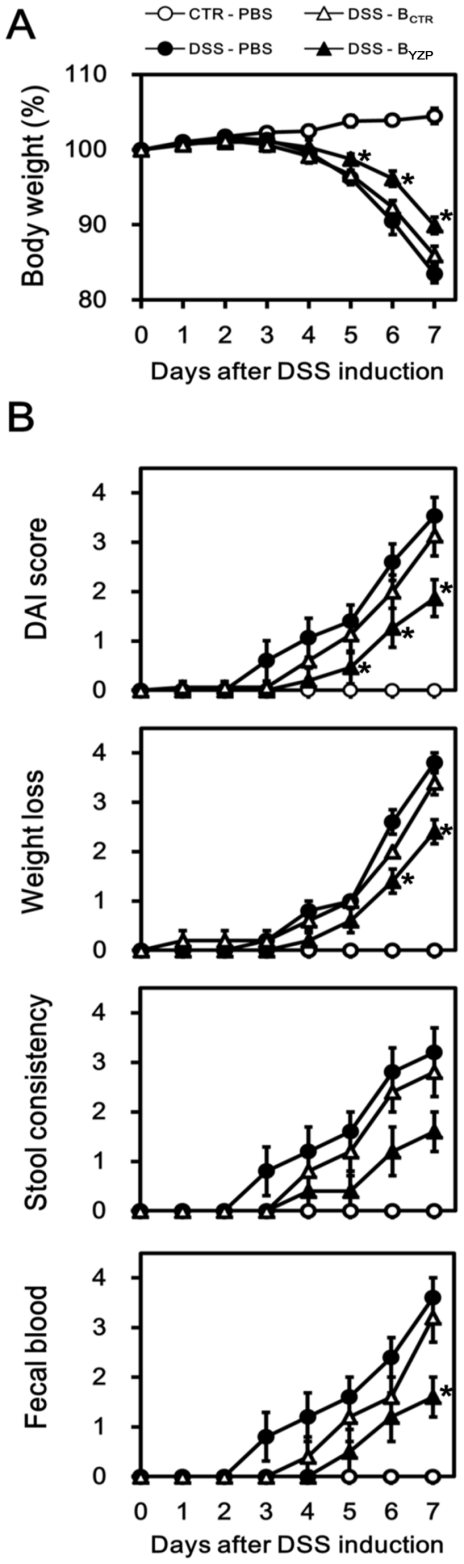
YZP-induced B cells alleviate disease activity in colitic mice. **A.** C57BL/6J mice were injected i.p. with PBS (0.2 mL; •), YZP-induced B cells (1.5×10^7^ in 0.2 mL PBS; ▴), or control B cells (1.5×10^7^ in 0.2 mL PBS; Δ) 1 day before 3% DSS induction. One group of control mice was administered PBS i.p., and the remaining mice ingested normal drinking water (○). The body weights of the mice were monitored daily, and the data are presented as the mean ± SD, where the asterisks indicate significant differences among DSS-ingested groups (*p*<0.05, n = 5). **B.** The disease activity index was scored based on the degree of weight loss, stool consistency, and fecal bleeding as detailed in Supporting Information Table S3 and was monitored daily. The data are presented as the mean ± SEM, and the asterisks indicate significant differences among DSS-ingested groups (*p*<0.05, n = 5).

**Figure 8:**
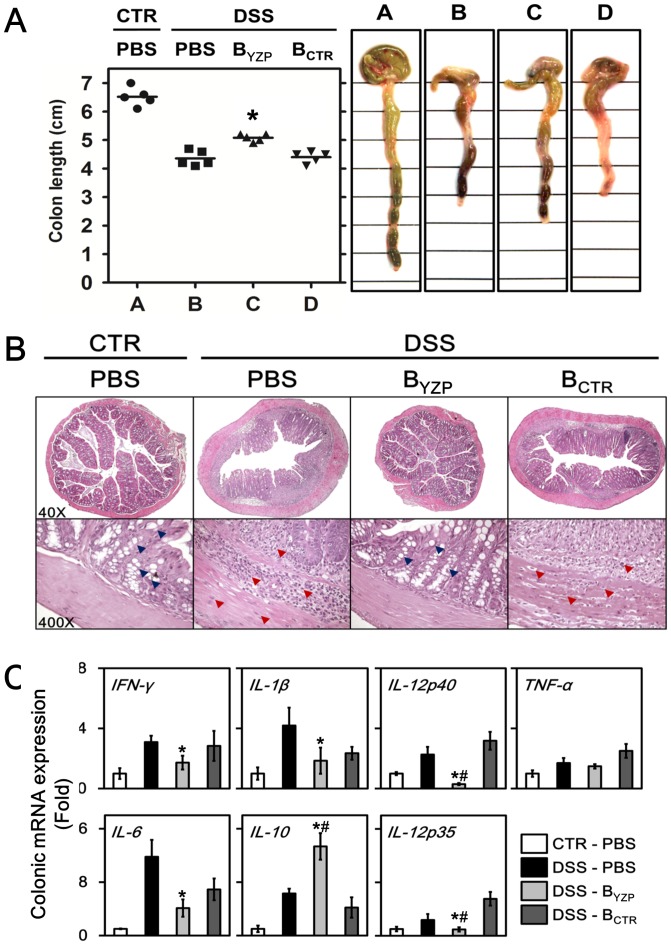
YZP-induced B cells ameliorate colonic damage in colitic mice. **A.** The colon lengths of healthy mice (•) and DSS-ingested mice that received i.p. PBS (▪), YZP-induced B cells (B_YZP_; ▴), or control B cells (B_CTR_; ▾) were measured. One representative colon sample from each group is shown in the right panel. The asterisk indicates significant differences among DSS-ingested groups (*p*<0.05, n = 5). **B.** Two colon samples from each group were fixed in 10% buffered formalin, and 5-μm-thick sections were cut and stained with H&E. H&E-stained cross-sections were examined with a microscope. One representative colon section is shown. The blue arrowheads indicate intact goblet cells, and the red arrowheads indicate infiltrated leukocytes. **C.** Total RNA was extracted from 3 colon samples from each group and reverse transcribed into cDNA for cytokine gene expression analysis. The data are presented as the mean ± SD (n = 3). The asterisks indicate significant differences (*p*<0.05) between DSS-ingested, PBS i.p. mice, and B_YZP_ i.p. mice. The # symbols indicate significant differences (*p*<0.05) between DSS-ingested, B_CTR_ i.p. mice, and B_YZP_ i.p. mice.

The mice began to lose weight 4 days after the administration of 3% DSS. Noticeably, the weight loss in mice receiving B-YZP was statistically less severe than those receiving B-CTR or PBS (**Figure 7A**). The disease activity index (DAI) scores of mice receiving B-YZP were also significantly lower than those of DSS-treated mice (**Figure 7B**). Reduction in colon length, which could be considered an indirect marker of colitis, was less severe in mice receiving B-YZP (**Figure 8A**). Histological assessment revealed that the colon segments from mice administered 3% DSS presented mucosal layer thickening, goblet cell disruption (blue arrowheads in **Figure 8B**), and severe leukocyte infiltration (red arrowheads in **Figure 8B**). Remarkably, the colon segments from mice receiving B-YZP displayed histological features comparable with those of healthy mice. Dysregulated colonic cytokine expression is a major symptom of chemical-induced intestinal inflammation [31]; hence, we examined the mRNA expression of the cytokines present in the colon samples. DSS administration upregulated the colonic gene expression of IFN-γ, IL-1β, IL-6, IL-12p35, and IL-12p40; however, the upregulation of inflammatory cytokines was drastically repressed after the adoptive transfer of B-YZP. Moreover, colonic gene expression of the regulatory cytokine IL-10 was significantly higher in mice receiving B-YZP than in DSS-treated mice (**Figure 8C**).

### The interaction between YZP and TLR4/TLR2 is involved in Breg induction

**Figure 9:**
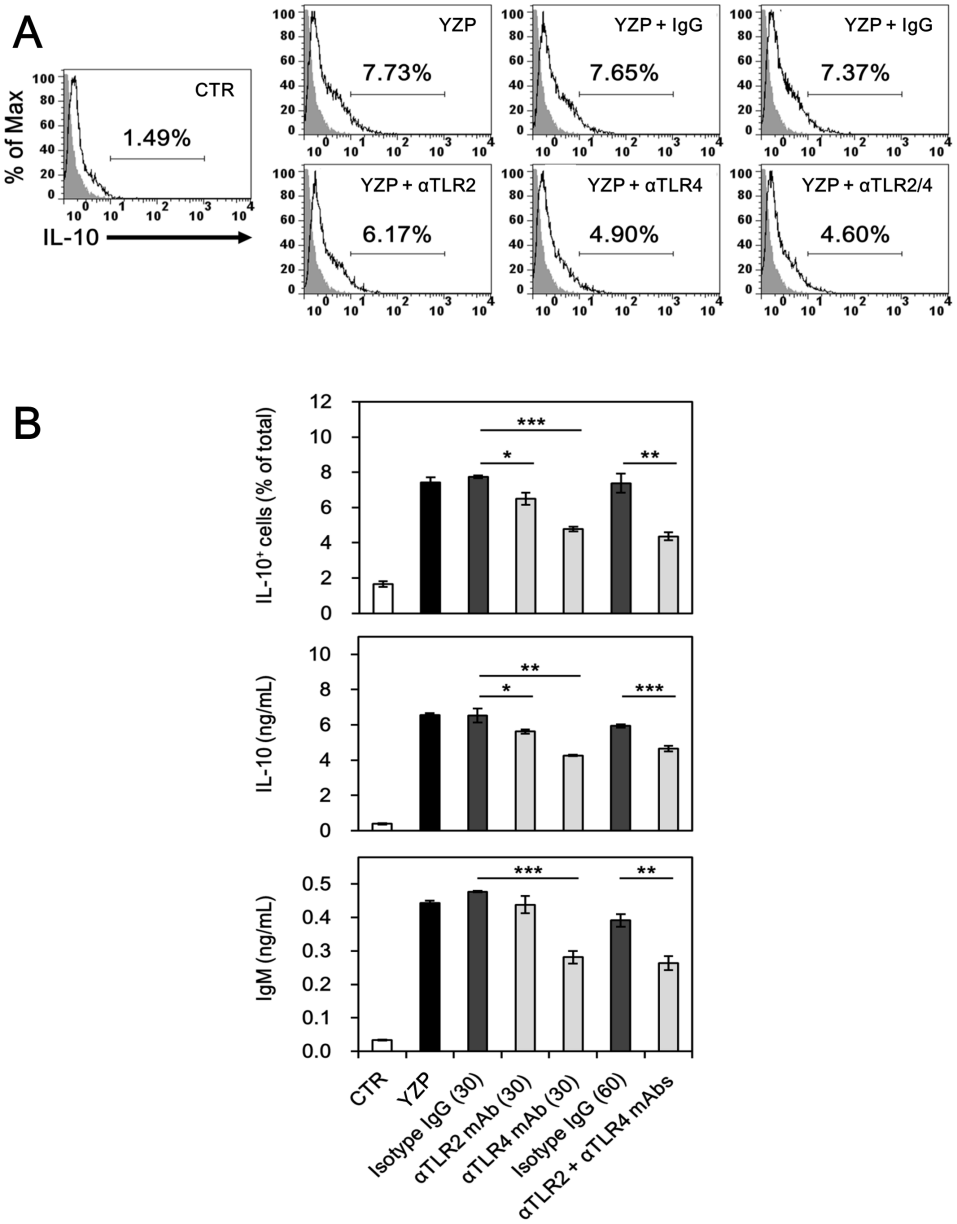
YZP-induced B cell activation is dependent on TLR2 and TLR4. **A.** MACS-purified CD19^+^ B cells were pre-incubated with isotype-matched IgG (30 or 60 μg/mL), anti-TLR2 mAb (30 μg/mL), anti-TLR4 mAb (30 μg/mL), or anti-TLR2 mAb plus anti-TLR4 mAb (30 μg/mL each) for 1 h. The cells were subsequently stimulated with YZP (10 μg/mL) for 48 h. One group was left untreated as a negative control, and one group was stimulated with YZP without antibody pre-incubation as a positive control. The cells were subsequently harvested for intracellular IL-10 staining. The numbers indicate the percentages of IL-10-positive cells among the total cells. **B.** The culture supernatant was collected for IL-10 and IgM quantification. The data are presented as the mean ± SD (n = 3). The asterisks indicate significant differences among groups (*, *p*<0.05;**, *p*<0.01; ***, *p*<0.005).

**Figure 10:**
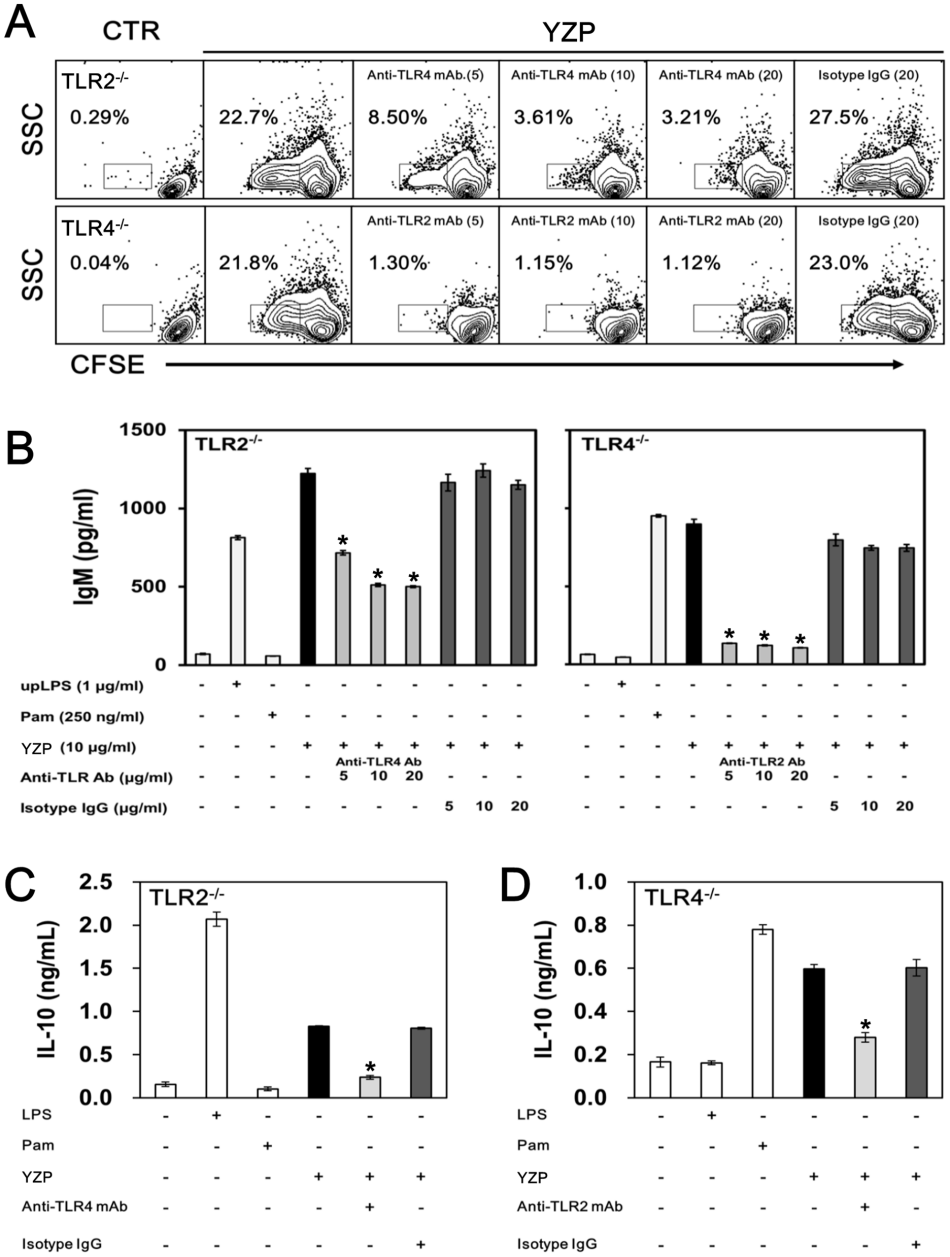
Blockages of both TLR2 and TLR4 were required to inhibit B cell differentiation induced through YZP. **A.** MACS-purified TLR2^–/–^ and TLR4^–/–^ CD19^+^ B cells were pre-incubated with isotype-matched IgG (30 μg/mL), anti-TLR2 mAb (30 μg/mL), or anti-TLR4 mAb (30 μg/mL) for 1 h and subsequently stimulated with YZP (10 μg/mL) for 48 h. One group was left untreated as a negative control, and one group was stimulated with YZP without antibody pre-incubation as a positive control. The cells were then harvested for CFSE staining cell proliferation analysis. **B.** The culture supernatant was collected, and the levels of secreted IgM and (**C** and **D**) IL-10 were measured. The data are presented as the mean ± SD (n = 3). The asterisks indicate significant differences (*p*<0.05) among YZP-treated groups.

Fungal proteins have been shown to modulate immune responses through TLR2 and/or TLR4 ligation [14], [29], [30], [32]. In addition, signal transduction mediated through TLRs is required for the development and function of Breg [33]. The use of neutralizing Abs to block TLR2 and/or TLR4 inhibited the ability of YZP to induce IL-10^+^ Bregs (**Figure 9A**); TLR4 neutralization caused approximately 40% of the reduction in IL-10 and IgM production, while TLR2 neutralization caused a 14% reduction in IL-10 production. When both TLR2 and TLR4 were blocked through Abs, the YZP-induced IL-10^+^ Breg population and secreted IL-10 and IgM were reduced by 41%, 22%, and 33%, respectively (**Figure 9B**). We used gene knockout mice to determine if the loss of the individual TLR2 or TLR4 genes abrogates the effects of YZP on B lymphocytes. B cells purified from TLR2^–/–^ and TLR4^–/–^ mice proliferated and secreted IgM in response to YZP (Figure S4). The application of TLR4- and TLR2-neutralizing Abs completely inhibited YZP function in TLR2^–/–^ and TLR4^–/–^ B cells, respectively (**Figure 10**). These results indicated that interactions with TLR2 and TLR4 might be crucial for the B cell-activating effect of YZP.

**Figure 11:**
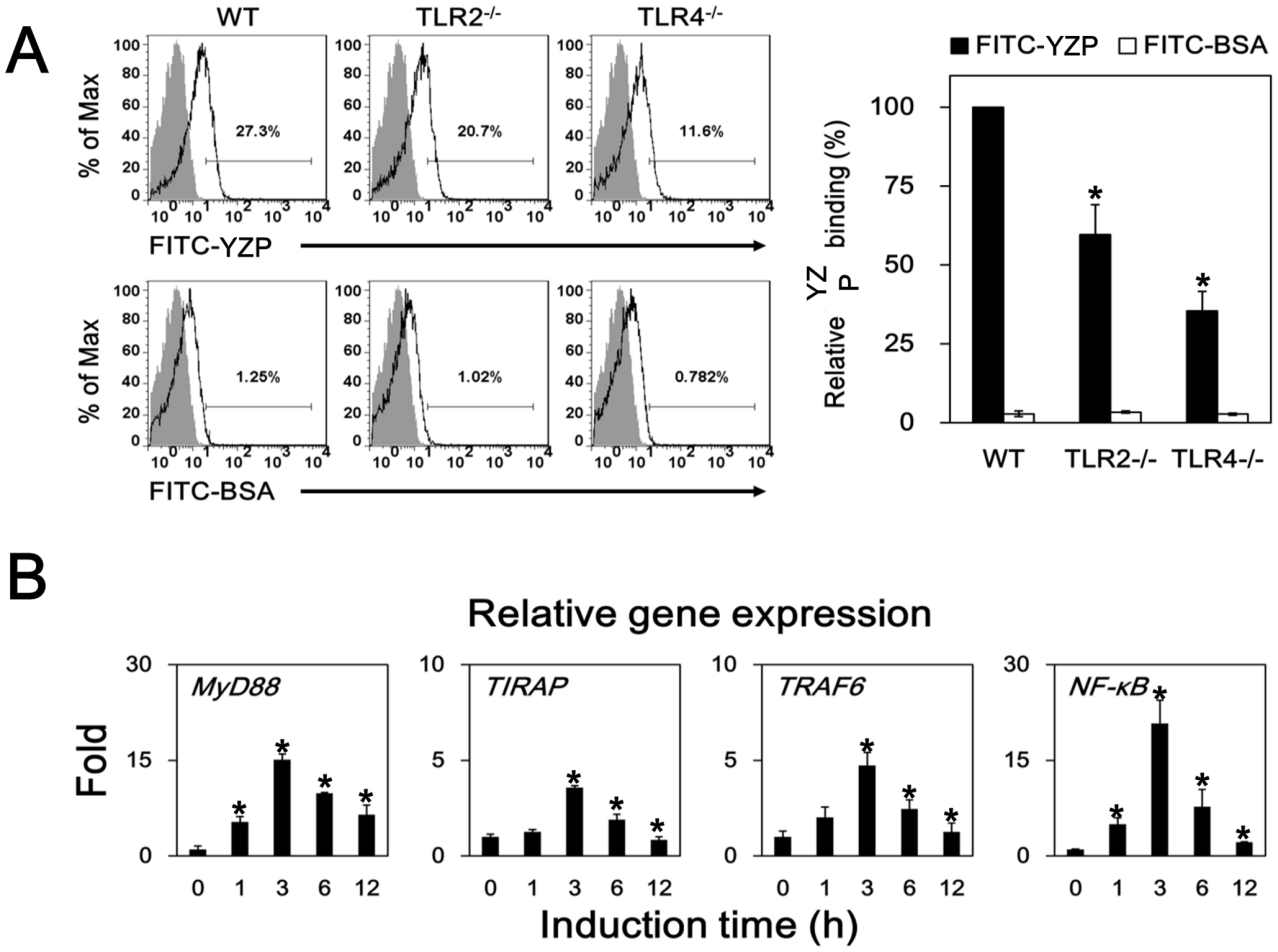
YZP binds to B cells through TLR2 and TLR4 and activates the gene expression of TLR-signaling molecules. **A.** MACS-purified CD19^+^ B cells from WT, TLR2^–/–^, and TLR4^–/–^ mice were pre-incubated with YZP (shaded grey), FITC-labeled YZP, or FITC-labeled BSA for 30 min. The cells were subsequently harvested for flow cytometry analysis. The numbers indicate the percentages of positive fluorescent cells among the total cells. The right panel shows the results from 3 independent experiments, presented as the mean ± SD (n = 3). The asterisks indicate significant differences (*p*<0.05) between TLR^–/–^ and WT mice. **B.** MACS-purified CD19^+^ B cells were stimulated with YZP (10 μg/mL) for the indicated periods. Total RNA was extracted and subsequently reverse transcribed to cDNA for gene expression analysis. The asterisks indicate that the data are significantly different (*p*<0.05) from the 0 h control group.

The interaction between YZP and B cells was studied in TLR2 and TLR4 gene knockout mice with FITC-labeled YZP. Splenic B cells from WT, TLR2^–/–^, and TLR4^–/–^ mice cultured with FITC-YZP or FITC-BSA were analyzed by flow cytometry. The populations of YZP^+^ cells in WT, TLR2^–/–^, and TLR4^–/–^ B cells were 27%, 21%, and 11%, respectively. Compared with WT B cells, the YZP^+^ cell populations were reduced 40% and 65% in TLR2^–/–^ and TLR4^–/–^ B cells, respectively (**Figure 11A**). These findings are consistent with the reduced YZP activity observed in TLR2/4-blocked B cell cultures (**Figure 9** and **Figure 10**). The expression of genes downstream of the TLR signaling pathway was studied by qPCR to determine if YZP activates B cells through TLR-mediated signals. Significant induction of MyD88, TIRAP, TRAF6, and NF-κB gene expression was observed within 3 h (**Figure 11B**), suggesting that the TLR signaling pathway is involved in the activation of B cells through YZP.

## Discussion

Despite extensive research on the pharmacological efficacies of *T. versicolor* extracts, studies on *T. versicolor* proteins have been scarce. In the present study, we demonstrated for the first time that YZP, a purified *T. versicolor* protein that is free of polysaccharides, exhibited extraordinary effects on the regulation of B-cell immune responses. Previously, it was reported that Krestin, a *T. versicolor* polysaccharopeptide extract, activates IgM secretion in the human B-cell line BALL-1 [5]. We discovered that Krestin also stimulated a relatively lower IL-10 production by murine splenic B cells as compared to YZP (Figure S5). However, Krestin comprises a mixture of polysaccharopeptides and 30% proteins [13]; it was difficult to ascertain which ingredients were responsible for the effect of this extract. Based on our results, it is reasonable to propose that YZP might greatly contribute to the activity of *T. versicolor* polysaccharopeptide extracts. Similar results have been observed for *Ganoderma lucidum*; the *G. lucidum* protein LZ-8 exhibits effects that both overlap with and differ from those of the *G. lucidum* polysaccharides PS-G, and the deproteination of PS-G hampers its immune-activating effect [16]. We also showed that Reishi-F3, a polysaccharide fraction isolated from *G. lucidum*, induced mild IL-10 production by B cells (Figure S5). This corresponded to the previous report demonstrating that Reishi-F3 activated B-cell differentiation in both murine and human [34].

NGS and de novo assembly are powerful technologies for genomic and transcriptomic research [20]–[24]. In the present study, we utilized these technologies and developed a cloning method that transcended the traditional rapid amplification of cDNA ends (RACE) approach. RACE cloning is based on PCR using primers degenerated from the N-terminal amino acids sequence and universal primers. This method provides a solid approach for cloning a new gene; however, because codon usage can differ among species and each amino acid can be encoded by up to 6 different codons, RACE cloning is a time-consuming and labor-intensive process. Therefore, we first identified the possible genes (the contigs) present in each species and then searched for the target gene, to reduce the time and labor typically spent on identifying adequate primer sets. We demonstrated the feasibility of this method in this study by identifying the YZP gene and confirming its fidelity through traditional PCR cloning techniques. Nevertheless, one possible disadvantage of this approach is the relatively high cost of an NGS run compared with that of classical RACE PCR; whether the saved time and labor compensates for the high cost should be cautiously considered. We also compared the amino acids sequence of YZP with other fungal immunomodulatory proteins and found that the sequence of YZP shared 56% of identical and 74% of similar amino acid residues with that of the immunomodulatory protein from *Taiwanofungus camphoratus* (Accession number: AAT11911.1; Figure S6).

Natural products, such as the *Anoectochilus formosanus* protein IPAF, Krestin, and Reishi-F3, activate IgM secretion in murine and human B cells [5, 15, and 34]. In the present study, we demonstrated that fungal proteins such as YZP exert B cell-stimulating effects beyond Ab secretion and that YZP induces the production of IL-6 and IL-10 in B cells. Studies have demonstrated that IL-6 exhibits immune-suppressive functions, such as inhibiting the production of TNF-α and controlling endotoxin-induced inflammation [35]–[37]. IL-10 is also a pluripotent regulatory cytokine that represses both Th1 [38] and Th2 [39] cytokine expression, and reduced secretion of inflammatory products by LPS-activated macrophages has been observed in vitro [40]. In our mixed leukocyte reaction, the IL-10 produced by YZP-induced B cells had significant effect on the suppression of inflammatory cytokine secretion.

We also revealed that YZP specifically induced the CD1d^+^ MZ B cells to secrete IL-10. This was consistent with previous researches showing that MZ B cells were the major IL-10 producing B cells among other B cell subsets [41]. In addition, we showed that YZP enhanced B-cell CD1d expression, which might be associated with the increase in IL-10 production [42].

The Breg subset characterized by increased CD1d expression was first identified in a mice model of chronic colitis in which CD1d^hi^ B cells produced IL-10 and suppressed the progression of intestinal inflammation through the repression of IL-1 and STAT3 activation in a CD1d-dependent manner [43]. In addition to IL-1β suppression, we demonstrated here that CD1d^hi^ IL-10-producing B cells also significantly inhibited IFN-γ and IL-12 expression in the colons of DSS-fed mice. The anti-colitis efficacy of a *T. versicolor* extract has also been demonstrated in an experimental colitis mice model, in which the oral administration of the extract suppressed the gene expression of STAT1, STAT6, IL-4, and IFN-γ [44]. Similar disease-alleviating effects were achieved through the adoptive transfer of CD1d^hi^CD5^+^ B cells into DSS-fed mice, in which the expression of CD19 and IL-10 is critical to the regulatory activities of these cells [45]. Bregs are a unique subset of B cells that can regulate immune-related diseases such as inflammation and autoimmunity [27], [28]. Based on experimental autoimmune diseases in which Bregs elicit significant protection against inflammatory pathologies, it has been proposed that activated Bregs might be used as cell-based immunotherapies for autoimmune diseases [46]. Currently, TLR ligands such as LPS and PGN are utilized for Breg-stimulation; however, these molecules might be unsuitable for therapeutic use due to their potential danger to the host. Because the endotoxin level in YZP was as low as 0.013 EU/μg, YZP might serve as a promising candidate for Breg stimulation.

MZ B cells and B1 cells are innate-like B cells that respond to TLR ligands. When activated through TLR signals, MZ B cells proliferate and differentiate into IgM-secreting cells, whereas B1 cells differentiate into IgA-secreting cells [47], [48]. Accordingly, YZP activated CD1d^+^ (MZ) B cells to differentiate into IgM- and IL-10-producing cells.

Interestingly, a recent study showed that CD1d^hi^CD5^+^ B10 cells maintain Ab secretion, even after transformation into IL-10-producing Bregs and that most B10 cells express high levels of IgM rather than other Igs [49]. Breg-produced Abs have also been proposed to facilitate pathogen clearance through opsonization or complement-mediated phagocytosis. In addition, the production of Abs also regulates inflammatory responses and suppresses experimental colitis [47], [50]. Therefore, in addition to IL-10, YZP-induced B cells secrete IgM, which contributes to the anti-inflammatory effects of these cells.

Since YZP principally induced the MZ B cells, an innate-like B cell subset that require TLR stimulation for activation [41], it was reasonable to discover that TLR signals were involved in YZP-induced Breg differentiation. We found that TLR4 was critical for YZP to activate IL-10 and IgM production by B cells, whereas TLR2 also participated to a lesser extent in B cell activation. However, these TLR are also ultilized by endotoxins such as LPS and PGN. To exclude the possibility of endotoxin contamination, we tested all our samples using the LAL assay and confirmed that endotoxin level were lower than 0.01 EU/μg, which equals to 0.001 ng/μg under our assay condition. The working dosages of YZP in this study were from 2.5 to 20 μg/mL, meaning that under the highest dosage, endotoxin contamination was as low as 0.02 ng/mL. Furthermore, we demonstrated the binding between YZP and TLR4/TLR2 by labeling the YZP with FITC, an amine specific fluorescent dye that reacts with protein rather than lipopolysaccharide or polysaccharides. Taken together, we exempted the noise of endotoxin contamination and demonstrated that YZP took advantage of the TLR signaling route to stimulate Breg activation. Nevertheless, although efforts have been made to validate the participation of TLR4 and TLR2 in the YZP-induced Breg differentiation, the interactions between these molecules are yet to be well-defined. It should be noticed that still other receptors and signal pathways might also be involved in the YZP-B cell interaction.

### Conclusions

We purified a new *T. versicolor* protein, YZP, and cloned the complete nucleotide sequence of the YZP gene. We developed an advanced method for gene cloning by combining RNA-seq and de novo assembly technologies. This novel approach greatly reduced the time and labor typically required for traditional RACE cloning. We observed that YZP activates IL-10-producing CD1d^+^ Breg differentiation and demonstrated the regulatory functions of YZP-induced B cells in vitro and in vivo. Moreover, we determined that Breg activation is induced by YZP through the TLR2/4-mediated signaling pathway and evaluated the immunotherapeutic potential of this protein against autoimmune diseases.

## Methods

### Purification of YZP from *T. versicolor* fruiting bodies

The fruiting bodies of dried *T. versicolor* (**Figure 1A**) were purchased from the Hsin Kan Yuan Chinese medicine pharmacy (Taipei, Taiwan) and were authenticated by Dr. Sheng-Hua Wu from The National Museum of Natural Science, Taichung, Taiwan. The fruiting bodies were weighed, ground to a powder with a grinder mill (Rong Tsong, Taipei, Taiwan), and subsequently immersed overnight in extraction buffer containing 5% (v/v) acetic acid, 0.1% (v/v) 2-mercaptoethanol, and 0.308 M sodium chloride. The *T. versicolor* powder solution was homogenized by sonication for 20 min on ice using a Sonicator XL2015 (MISONIX, Farmingdale, NY, U. S. A.), and the homogenates were filtered through Glass-Microfiber Discs (Sartorius Stedim Biotech, Aubagne, France) to remove debris. The crude proteins were precipitated after the addition of 90% saturated ammonium sulfate (603 g/L) to the filtered homogenates. The homogenate/ammonium sulfate solution was allowed to stand for at least two days for precipitation. The crude proteins were obtained by centrifugation at 3500×g for 30 min at 4°C in a refrigerated Allegra 22R Centrifuge (Beckman Coulter, Brea, CA, U. S. A.). The precipitates were resuspended in 0.02 M Tris buffer (TB, pH 8.2) and successively filtered through 0.45- and 0.20-μm polyethersulfone (PESU) membranes (Sartorius Stedim Biotech) prior to ion exchange chromatography.

The crude protein solution was fractionated on an AKTA fast protein liquid chromatography (FPLC) system equipped with a HiTrap Q anion exchange column (GE Healthcare, Buckinghamshire, U. K.) pre-equilibrated with TB. The crude protein solution was loaded onto the column, and the unbound sample was eluted with two column volumes of TB. Subsequently, the column was eluted with a linear gradient of TB containing 0.15 to 0.16 M NaCl, and the column was washed and regenerated with 0.5 M NaCl Tris buffer. The protein profile in the fractions was visualized by SDS-PAGE, followed by staining with Coomassie Brilliant Blue. The fractions enriched with YZP were pooled and re-dialyzed into TB for further purification. The YZP-enriched fraction was loaded onto a Resource Q anion exchange column (GE Healthcare) pre-equilibrated with TB. The column was eluted with a linear gradient of TB containing 0 to 0.15 M NaCl. The fractions containing purified YZP were collected and dialyzed into phosphate buffered saline (PBS) containing 1.47 mM KH_2_PO_4_, 0.137 M NaCl, 2.68 mM KCl, and 8.10 mM Na_2_HPO_4_. Endotoxin contamination in the purified YZP samples was always less than 0.013 EU/mg, as tested with a ToxinSensor™ Chromogenic LAL Endotoxin Assay Kit according to the manufacturer's protocol (GenScript Inc., Piscataway, NJ, U. S. A.).

### SDS-PAGE and molecular weight determination of YZP

The crude protein extracts, YZP-enriched fractions, non-YZP fractions, and purified YZP were pretreated with an appropriate volume of 5X sodium dodecyl sulfate (SDS) sample buffer (100 mM Tris-HCl, 4% SDS, 20% glycerol, 10% 2-mercaptoethanol, and 100 mM DTT, pH 6.8). The samples were boiled for 10 min and analyzed with a Bio-Rad mini protein III gel apparatus (Bio-Rad). The gels were visualized by staining with Coomassie Brilliant Blue R250 or periodic acid-Schiff (PAS) reagent for carbohydrate determination. The MW of YZP was calculated based on the band migration relative to the PageRuler Prestained Protein Ladder #SM0671 (Fermentas, MD, U. S. A.).

### Illumina sequencing

Total RNA was extracted from 1 g of fresh *T. versicolor* fruiting bodies with a Total RNA Miniprep Purification Kit (GMbiolab, Taipei, Taiwan) according to the manufacturer's protocol. The concentration and absorbance 260/280 ratio of the RNA samples were measured with a NanoDrop 1000 spectrophotometer (Thermal Scientific, Wilmington, DE, U. S. A.). RNA samples with absorbance 260/280 ratios above 2 were subjected to quality and integrity testing on an Agilent 2100 Bioanalyzer (Agilent Technologies, Santa Clara, CA, U. S. A.), and samples with RIN scores greater than 8.5 were used to construct the cDNA library with an Illumina mRNA sequencing sample preparation kit (Illumina, San Diego, CA, U. S. A.) according to previously described methods [21]. Briefly, the RNA samples were treated with DNase I, and poly-A mRNA was purified with poly-T oligo-attached magnetic beads. Poly-A mRNA fragmentation was performed using heat and divalent cation treatments. Double-stranded cDNA was synthesized with the fragmented mRNA as the template and ligated with an Illumina PE adaptor oligo mix. The mRNA-adaptor molecules of 200±25 bp were purified through gel extraction and used as templates for 15 cycles of PCR amplification. The amplified cDNA libraries were subsequently sequenced on an Illumina sequencing platform (GAII), and the raw reads were obtained using Solexa GA pipeline 1.6.

### De novo assembly of contigs and YZP gene identification

Prior to de novo assembly, the raw reads data obtained from the Illumina sequencing were processed to remove adaptor sequences, reads with ambiguous N bases, and duplicated sequences. Using the default setting of CLC Genomics Workbench 5 (Cambridge, MA, U. S. A.), de novo contig assembly was performed by combining the processed-reads based on sequence overlap. To identify the gene encoding YZP, the contigs were translated into protein sequences, which were subsequently matched to the N-terminal amino acid sequence of the YZP derived using previously described methods [15]. The YZP gene was PCR-cloned using primers designed based on the sequence of the contig encoding the N-terminus of YZP (Table S1 and Table S2).

### Mice and cell cultures

C57BL/6J and BALB/cByJ mice ranging between 6 and 8 weeks of age, were purchased from the National Laboratory Animal Center Taipei, Taiwan. TLR2 (B6.129-*Tlr2^tm1Kir^*/J) and TLR4 (C57BL/10ScNJ) gene knockout mice were purchased from The Jackson Laboratory. All mice were maintained in our animal facility under pathogen-free conditions, and all animal studies were approved and performed according to the guidelines of The Institutional Animal Care and Use Committee (IACUC) of National Taiwan University (Approval ID: NTU-IACUC-98-112).

Mice splenic cells were acquired as previously described [15]. Briefly, the spleens were excised from euthanized mice, and the total splenocytes and splenic CD19^+^ B cells or CD90^+^ T cells were purified using MACS system (Miltenyi Biotec, Bergisch Gladbach, Germany). The cells were then washed with PBS and suspended in RPMI medium (Hyclone, Logan, UT, U. S. A.) containing 10% fetal bovine serum (GIBCO-BRL Life Technologies, New York, NY, U. S. A.). The cells were cultured in 12-well flat bottom plate (Corning, Lowell, MA, U. S. A.) at 2.5×10^6^ cells/mL in a Heracell Incubator (Heraeus group, Hanau, Germany) at 37°C and 5% CO_2_.

### Flow cytometry analysis

The cells were harvested and washed twice with FACS buffer containing 2% (v/v) FBS and 0.1% (w/v) sodium azide dissolved in PBS. The cells were suspended in FACS buffer and incubated with Abs on ice for 30 min at a concentration recommended by the manufacturers. The following Abs were used in this study: FITC-labeled anti-mouse CD40, anti-mouse CD45R/B220, anti-mouse CD80, anti-mouse CD86, and anti-mouse MHC-II (eBioscience, San Diego, CA, U. S. A.); phycoerythrin (PE)-labeled anti-mouse CD3, anti-mouse CD5, anti-mouse CD23, anti-mouse CD25, and anti-mouse CD69 (eBioscience); FITC-labeled anti-mouse CD1d and PE-labeled anti-mouse CD138 (BD Pharmingen, San Diego, CA, U. S. A.) and PerCP-Cy5.5-labeled anti-mouse CD5 (eBioscience). Data were acquired on a FACScan (BD Bioscience, San Diego, CA, U. S. A.), and the results were analyzed with FlowJo software (Tree Star, Ashland, OR, U. S. A.).

### Detection of intracellular IL-10 production

Splenocytes or MACS-purified CD19^+^ splenic B cells were cultured with YZP (20 μg/mL) for 48 h. During the last 5 h, phorbol 12-myristate 13-acetate (PMA; 50 ng/mL; Sigma-Aldrich, St. Louis, MO, U. S. A.), ionomycin (500 ng/mL; Sigma-Aldrich), and monensin (2 μM; eBioscience) were applied to the cell culture to facilitate intracellular staining. The cells were subsequently harvested, and staining for surface markers was performed as previously described. After surface staining, the cells were washed twice with FACS buffer and then permeated and fixed with Cytofix/Cytoperm solution (BD Bioscience) for 20 min on ice. The cells were subsequently washed twice with Perm/Wash solution (BD Bioscience) and stained with PE-labeled or PerCP-Cy5.5-labeled anti-mouse IL-10 (eBioscience) for 30 min on ice. The excess antibodies were removed by washing with Perm/Wash solution, and the cells were analyzed by flow cytometry.

### Measurement of secreted cytokines and immunoglobulins

MACS-purified CD19^+^ B cells were stimulated with YZP (0-20 μg/mL) for 72 h, and the cell-free culture supernatant was collected for cytokine and immunoglobulin quantification. OptEIA murine IL-2, IL-4, IL-5, IL-6, IL-10, IL-13, TNF-α, and IFN-γ ELISA kits (eBioscience) and Mouse IgM and Mouse IgG ELISA quantification kits (Bethyl Laboratories, Inc., Montgomery, TX, U. S. A.) were used according to the manufacturer's instructions, and the results were acquired by measuring the absorbance at 450 nm in a Bio-Rad 3550-UV microplate reader. The cytokine concentration was calculated based on the calibration curve established using the standard curve.

### In vitro immunosuppressive assay of YZP-stimulated B cells

MACS-purified CD19^+^ splenic B cells were stimulated with 20 μg/mL YZP (B-YZP) or 20 μL PBS (B-CTR) for 48 h. The cells were subsequently washed with fresh medium to remove YZP. Peritoneal macrophages were obtained from mice injected i.p. with 1.8 mL of autoclaved thioglycolate (3%) 4 days before the experiment. The mice were euthanized, and the peritoneal cells were acquired via lavage of the peritoneal cavity with 10 mL PBS. The cells were cultured for 2 h, and the non-adherent cells were subsequently removed. The adherent macrophages were stimulated with LPS (1 μg/mL) for 12 h. The LPS-stimulated macrophages (MΦ-LPS; 2×10^6^) were washed and further co-cultured with 2×10^6^ B-YZP or B-CTR for 12 h. In some experiments, the macrophages were pre-treated with Ultra-LEAF™ Purified anti-mouse CD210 (IL-10 R) Antibody (BioLegend, San Diego, CA, U. S. A.) or Functional Grade Purified Rat IgG1, κ Isotype Ctrl (eBioscience) for 30 min prior to the mix leukocytes reaction. Different macrophage to B cell ratios were assessed by co-culturing 2×10^6^ MΦ-LPS with 0, 0.5, 1, 2, and 4×10^6^ B-YZP. The supernatant from the mixed leukocyte culture was collected to quantify TNF-α and IL-1β production.

### Induction and evaluation of DSS-induced intestinal injury

Six-week old male C57BL/6J mice were used for the experimental colitis model. The mice were randomly distributed into 4 groups (n = 5) and acclimatized for 1 week in our animal facility before DSS administration. The induction and evaluation of DSS-induced intestinal injury was modified from the previously described methods of Yanaba et al. [40]. For colitis induction, normal drinking water was replaced with sterilized reverse osmotic water containing 3% (w/v) dextran sulfate sodium (DSS, molecular mass 36 to 50 kDa, MP Biomedicals, OH, U. S. A.). The water consumption per mouse was monitored daily and was comparable among the groups. To evaluate DSS-induced intestinal injury, the disease activity index (DAI) was determined based on weight loss, stool consistency, and fecal bleeding (Table S3). On day 7 after DSS induction, the mice were euthanized to assess colonic damage. The colons were removed from the mice, and colon length was measured as an indirect marker of intestinal injury. The colons were cleaned, and the stool was flushed from each sample with ice-cold PBS. The colon samples were segmented for RNA extraction and histological analysis. For histological analysis, the colon segments were fixed in 10% buffered formalin. After paraffin embedding, 5 μm-thick cross sections were cut and stained with H&E and observed microscopically. The degree of intestinal injury was determined based on leukocyte infiltration, crypt damage, wall thickening, and loss of goblet cells.

### Quantification of colonic gene expression by real-time qPCR

For colonic gene expression analysis, 100 mg of mice colon tissue was lysed with 1 mL TRIzol reagent (Invitrogen). Total RNA was extracted following the manufacturer's instructions. Briefly, the tissue lysates were mixed thoroughly and subsequently centrifuged at 11,200×g for 15 min at 4°C. The aqueous phase was transferred to a new RNase-free Eppendorf tube, and an equal volume of absolute ethanol (molecular biology grade, Sigma-Aldrich) was added. The RNA was pelleted by centrifugation at 11,200×g for 15 min at 4°C. The RNA pellets were washed twice by adding 75% ethanol and centrifuging at 8,800×g for 5 min at 4°C. The RNA was subsequently resuspended in 20 μL RNase-free water and allowed to dissolve for 10 min at 55°C. Approximately 1 to 5 μg of RNA was added to a RNA to cDNA EcoDry™ premix oligo dT (Clontech, CA, U. S. A.) and reversed transcribed into first-strand cDNA at 42°C for 1 h, followed by incubation at 70°C for 10 min. The cDNA product was utilized as the template for gene expression analysis. The differential expression of individual genes was assessed by qPCR on a MyiQ Single Color Real-Time PCR Detection System (Bio-Rad). The housekeeping genes GAPDH or β-actin were used to confirm the quality of the cDNA in the qPCR analysis. The expression of the tested genes was quantified according to the cycling threshold (Ct) analysis with iQ5 software (Bio-Rad). The primers used in this study are listed in Table S1 and Table S2.

### Investigating TLR2/4 involvement in YZP-induced B cell activation

The MACS-purified splenic CD19^+^ B cells acquired from wild type, TLR2^–/–^, and TLR4^–/–^ mice were used to study the function of TLR2 and TLR4 in YZP-induced B cell activation. The cells were blocked with 5 to 20 μg/mL of functional grade purified anti-mouse TLR4/MD-2 complex, functional grade purified anti-human/mouse CD282, or isotype-matched IgG for 1 h. In some experiments, the cells were labeled with CFSE before blocking (the detailed method for CFSE staining is described in Methods S1). Subsequently, the cells were washed and resuspended in fresh medium and stimulated with YZP (10 μg/mL) for 48 h. Cell proliferation, IgM production, and intracellular and secreted IL-10 production were detected as previously described.

### Statistical analysis

All in vitro experimental data are presented as the mean ± SD of a representative result from at least 3 independent experiments performed in triplicate (n = 3). The data for the DSS-induced murine colitis model represent a single result obtained from 3 independent animal experiments performed in pentuplicate (n = 5). One-way ANOVA was performed for statistical comparisons using a SAS software (Statistical Analysis System, SAS Institute, U. S. A.). Differences among the experimental data were deemed significant when a *p*-value below 0.05 was obtained.

## Supporting Information

Figure S1 **Immunomodulatory activities of the isolated proteins through various chromatography stages. A.** Murine peritoneal macrophages were cultured in the presence of the crude proteins extract, YZP-enriched fraction, and non-YZP fraction at indicated dosages for 24 h. The cell-free culture supernatant was then harvested for ELISA quantification of the secreted TNF-α. **B.** Murine splenocytes were cultured in the presence of the crude proteins extract, YZP-enriched fraction, and non-YZP fraction at indicated dosages for 72 h. The cellular enzyme activity was then assessed by tetrazolium dye (MTT) colorimetric assay.
(TIF)

Figure S2 **YZP activated cell proliferation and IL-10 production in murine splenic cells.**
**A.** Murine splenocytes, MACS-purified CD90^+^ T cells and MACS-flow-through CD90^–^ non-T cells were stimulated with indicated dosages of YZP for 72 h, and then the cell proliferation was assessed by BrdU ELISA. **B.** Murine splenocytes were stimulated with YZP of indicated dosages for 72 h (upper panel) or 10 μg/mL of YZP for indicated periods (lower panel), and IL-10 presented in the culture supernatant was quantified by ELSA. **C.** Murine splenocytes, MACS-purified CD19^+^ B cells and CD90^+^ T cells were stained with CFSE and stimulated with YZP (10 μg/mL) or conA (5 μg/mL) for 72 h, and then the cell proliferation was assessed by flow cytometry.
(TIF)

Figure S3 **Signal peptide and molecular weight prediction of YZP.**
**A.** The presence of putative signal peptide and cleavage site in YZP protein was predicted by submitting the complete amino acid sequence of YZP to the SignalP 4.0 Server (http://www.cbs.dtu.dk/services/SignalP/). **B.** The theoretical molecular weight of YZP was predicted by submitting the amino acid sequence of YZP without the putative signal peptide to the ExPASy – Compute pI/Mw tool (http://web.expasy.org/compute_pi/).
(TIF)

Figure S4 **Cell proliferation and IgM production of YZP-induced B cells from WT, TLR2^–/–^ and TLR4^–/–^ mice. A.** CFSE cell proliferation analysis of splenocytes from WT mice and CD19^+^ B cells purified from TLR2^–/–^ and TLR4^–/–^ mice. **B.** Quantification of secreted IgM production by CD19^+^ B cells purified from WT, TLR2^–/–^ and TLR4^–/–^ mice.
(TIF)

Figure S5 **Comparison of the B-cell IL-10-inducing activity of fungal proteins and polysaccharides.** MACS-purified murine splenic CD19^+^ B cells were cultured in the presence of YZP, Krestin, LZ-8, and Reishi-F3 at indicated dosages for 48 h. The cell-free culture supernatant was collected for IL-10 quantification by ELISA. Krestin was purchased from Kureha Corporation (Tokyo, Japan), LZ-8 was prepared in our lab as previously described [16], and Reishi-F3 was kindly provided by Dr. Hsien-Yeh Hsu from National Yang Ming University.
(TIF)

Figure S6 **Amino acid sequence alignment of YZP and the** ***Taiwanofungus camphoratus*** **immunomodulatory protein.** The amino acid sequence of YZP was analyzed using the BLAST tool on the website of NCBI (http://www.ncbi.nlm.nih.gov/) in search of proteins consisting of similar sequences to YZP and possess immuno-modulating functions. The amino acid sequence alignment of YZP and the immunomodulatory protein from *Taiwanofungus camphoratus* is shown.
(TIF)

Table S1 **Sequences of primers designed for PCR cloning of YZP gene.**
(DOCX)

Table S2 **Nucleotide sequences of the primers used in real-time qPCR.**
(DOCX)

Table S3 **Clinical scoring for disease activity index of colitis.**
(DOCX)

Method S1 **BrdU ELISA and CFSE cell proliferation analysis.**
(DOCX)
