# Supplementary figures and images for: Trametes versicolor Protein YZP Activates Regulatory B Lymphocytes – Gene Identification through De Novo Assembly and Function Analysis in a Murine Acute Colitis Model
Source: PLoS One. 2013 Sep 3;8(9):e72422. doi: 10.1371/journal.pone.0072422 (PMC3760908; doi:10.1371/journal.pone.0072422)

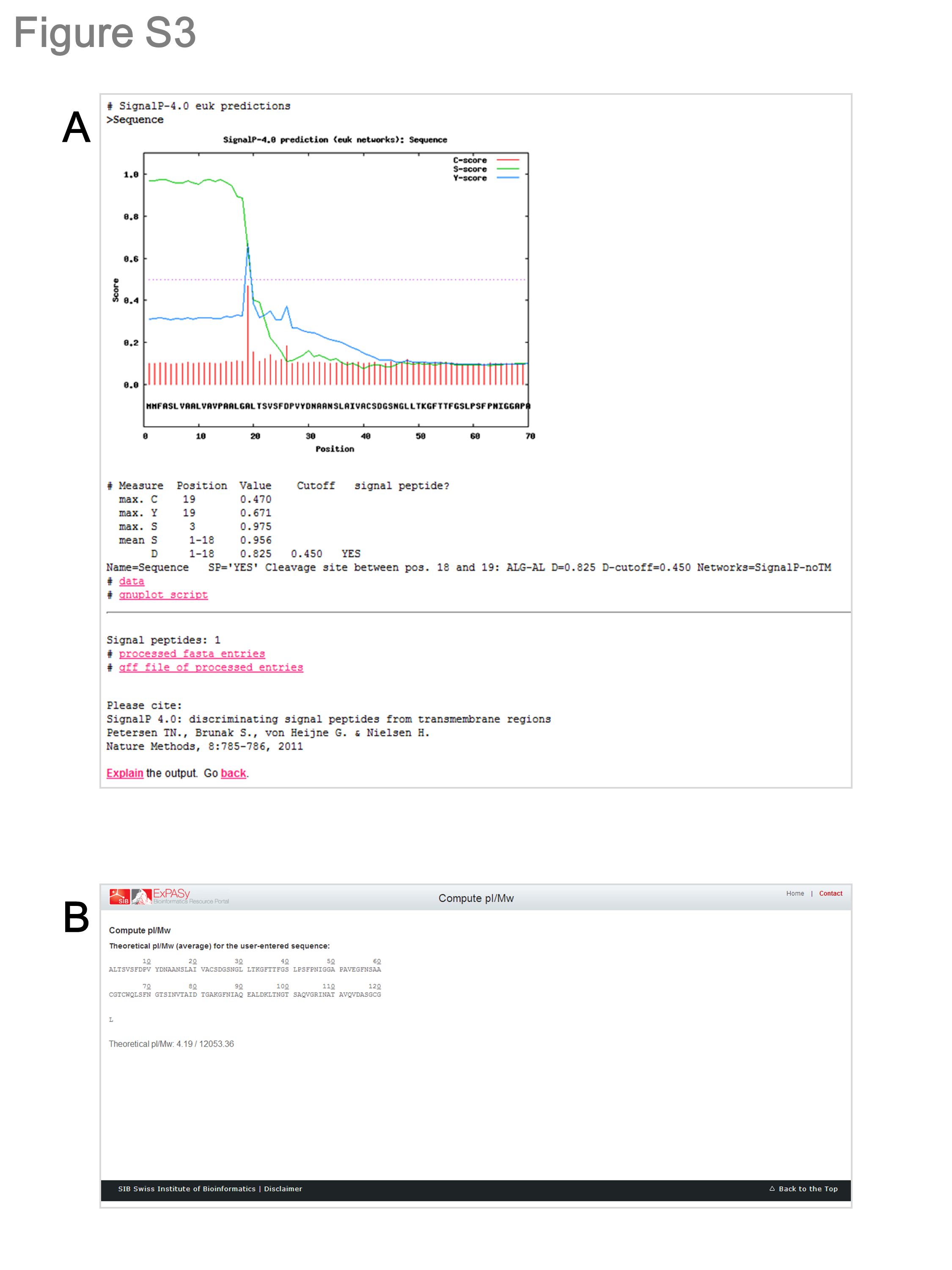

Supplement: Figure S3 — Signal peptide and molecular weight prediction of YZP. A. The presence of putative signal peptide and cleavage site in YZP protein was predicted by submitting the complete amino acid sequence of YZP to the SignalP 4.0 Server (http://www.cbs.dtu.dk/services/SignalP/). B. The theoretical molecular weight of YZP was predicted by submitting the amino acid sequence of YZP without the putative signal peptide to the ExPASy – Compute pI/Mw tool (http://web.expasy.org/compute_pi/). (TIF) [file pone.0072422.s003.tif]

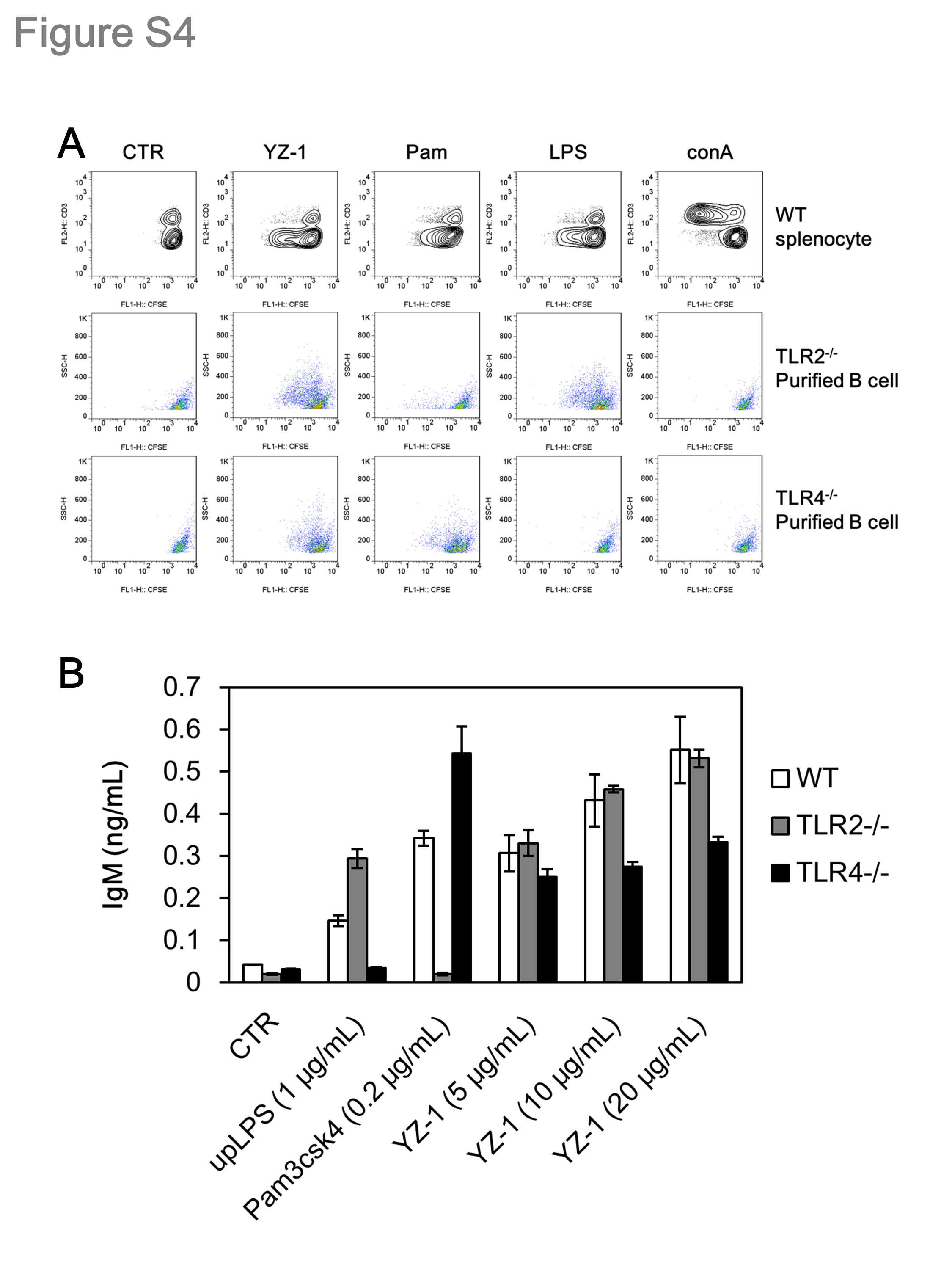

Supplement: Figure S4 — Cell proliferation and IgM production of YZP-induced B cells from WT, TLR2–/– and TLR4–/– mice. A. CFSE cell proliferation analysis of splenocytes from WT mice and CD19+ B cells purified from TLR2–/– and TLR4–/– mice. B. Quantification of secreted IgM production by CD19+ B cells purified from WT, TLR2–/– and TLR4–/– mice. (TIF) [file pone.0072422.s004.tif]

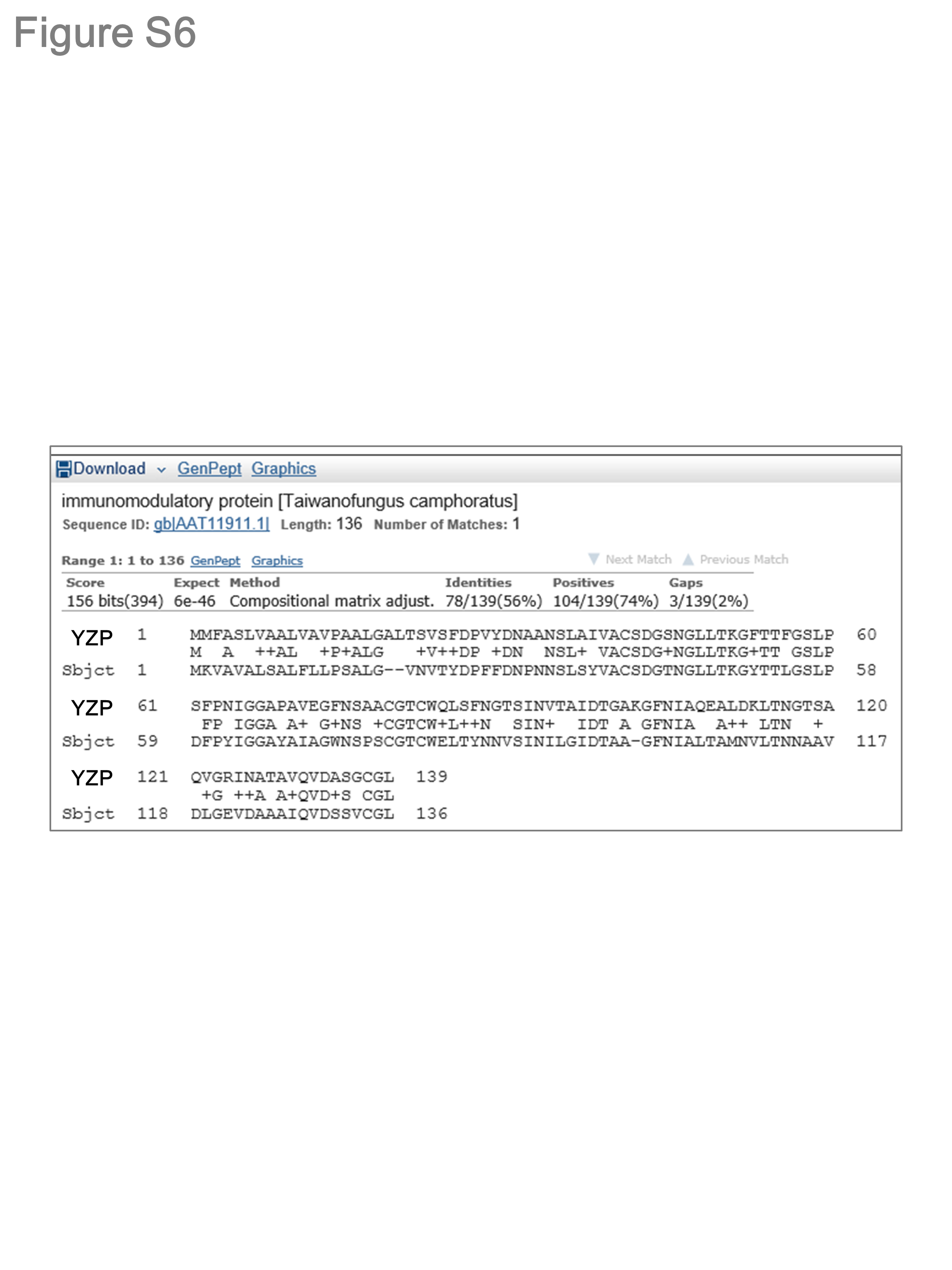

Supplement: Figure S6 — Amino acid sequence alignment of YZP and the Taiwanofungus camphoratus immunomodulatory protein. The amino acid sequence of YZP was analyzed using the BLAST tool on the website of NCBI (http://www.ncbi.nlm.nih.gov/) in search of proteins consisting of similar sequences to YZP and possess immuno-modulating functions. The amino acid sequence alignment of YZP and the immunomodulatory protein from Taiwanofungus camphoratus is shown. (TIF) [file pone.0072422.s006.tif]
