# Supplementary material for: Trametes versicolor Protein YZP Activates Regulatory B Lymphocytes – Gene Identification through De Novo Assembly and Function Analysis in a Murine Acute Colitis Model
Source: PLoS One. 2013 Sep 3;8(9):e72422. doi: 10.1371/journal.pone.0072422 (PMC3760908; doi:10.1371/journal.pone.0072422)
Supplement: Table S1 — Sequences of primers designed for PCR cloning of YZP gene. (DOCX) [file pone.0072422.s007.docx]

Table S1. Sequences of primers designed for PCR cloning of YZP gene.

|  | Nucleotide sequence of | |
| --- | --- | --- |
|  | Forward primer (5’ to 3’) | Reverse primer (5’ to 3’) |
| YZP1 | ACCTCCACCTCTGTTCAT | ATGAAGCGTCCCAGAATG |
| YZP2 | CGACCCAGTCTACGACAA | GTTAATCCTTCCCACCTGT |
| YZP3 | GTTCACCACGTTCGGATC | GCGATGTTGAAGCCCTTT |
