# Supplementary material for: Trametes versicolor Protein YZP Activates Regulatory B Lymphocytes – Gene Identification through De Novo Assembly and Function Analysis in a Murine Acute Colitis Model
Source: PLoS One. 2013 Sep 3;8(9):e72422. doi: 10.1371/journal.pone.0072422 (PMC3760908; doi:10.1371/journal.pone.0072422)
Supplement: Table S2 — Nucleotide sequences of the primers used in real-time qPCR. (DOCX) [file pone.0072422.s008.docx]

Table S2. Nucleotide sequences of the primers used in real-time qPCR.

|  | Nucleotide sequence of | |
| --- | --- | --- |
|  | Forward primer (5’ to 3’) | Reverse primer (5’ to 3’) |
| β-actin | GTGGGCCGCTCTAGGCACC | CTCTTTGATGTCACGCACGA |
| G3PDH | ATGAATACGGCTACAGCA | TGGAAATTGTGAGGGAGAT |
| IFN-γ | TGAGACAATGAACGCTACAC | CTTCCACATCTATGCCACT |
| IL-1β | CAGTGGTCAGGACATAATGG | GTCTTGGCCGACCACTAA |
| IL-2 | GTAAAACTAAAGGGCTCTGAC | TTGAGGGCTTGTTGAGAT |
| IL-4 | GGATTTGTTAGCATCTCTTGA | ATATGGCTCCTGGTACATTC |
| IL-5 | TGTTGACAAGCAATGAGACG | TGACAGGTTTTGGAATAGC |
| IL-6 | TGTTCTCTGGGAAATCGTG | AGTTTGGTAGCATCCATCA |
| IL-10 | AATAAGCTCCAAGACCAAGG | CAGACTCAATACACACTGC |
| IL-12p35 | TGCCTGGTAGCATCTAT | CAGAGTCTCGCCATTATGA |
| IL-12p40 | TCTCCCTCAAGTTCTTTGT | GCATTGGACTTCGGTAGAT |
| IL-13 | ATGGTATGGAGTGTGGAC | GGCTACTTCGATTTTGGTAT |
| LT-α | GGGAAGGTTGACTATTTATGG | CTTAGATGGGTCCTGTCTGA |
| NF-κB | CGGGATGGCTACTATGAG | AACCCGATTGATGAGCC |
| MYD88 | TATCTGCTACTGCCCCA | AGTCAGCTTCGTCAGG |
| Prdm1 | TCCTCTTCCTCTTCCTCTTC | AACAGGTCAGTAAGGCTCTT |
| TIRAP | AACCCGATTGATGAGCC | ATTCTCGACCAGTCTGAAG |
| TNF-α | CCTCCTCTTTTGCTTATGTT | CAATTACAGTCACGGCTC |
| TRAF6 | ATTCTCGACCAGTCTGAAG | ATGAAGGTTCCCTGTCT |
