# Supplementary material for: Trametes versicolor Protein YZP Activates Regulatory B Lymphocytes – Gene Identification through De Novo Assembly and Function Analysis in a Murine Acute Colitis Model
Source: PLoS One. 2013 Sep 3;8(9):e72422. doi: 10.1371/journal.pone.0072422 (PMC3760908; doi:10.1371/journal.pone.0072422)
Supplement: Table S3 — Clinical scoring for disease activity index of colitis. (DOCX) [file pone.0072422.s009.docx]

Table S3. Clinical scoring for disease activity index of colitis

| Score | Weight loss | Stool consistency | Fecal blood |
| --- | --- | --- | --- |
| 0 | <1% | normal stools | no bleeding |
| 1 | 1-5% | NA | NA |
| 2 | 6-10% | loose stools | Visible blood in stool |
| 3 | 11-15% | NA | NA |
| 4 | >15% | diarrhea | gross anus bleeding |

NA indicates that the scoring does not apply to this parameter.
