# Supplementary material for: Trametes versicolor Protein YZP Activates Regulatory B Lymphocytes – Gene Identification through De Novo Assembly and Function Analysis in a Murine Acute Colitis Model
Source: PLoS One. 2013 Sep 3;8(9):e72422. doi: 10.1371/journal.pone.0072422 (PMC3760908; doi:10.1371/journal.pone.0072422)
Supplement: Method S1 — BrdU ELISA and CFSE cell proliferation analysis. (DOCX) [file pone.0072422.s010.docx]

**Method S1. BrdU ELISA and CFSE cell proliferation analysis**

Splenic cells were seeded in 96-well U-bottom plate at 2.5×10^6^ cell/mL (0.1 mL/well) and stimulated with YZP (20 μg/mL) for 72 h. In the last 24 h, 10 μL of BrdU labeling reagent (100 μM) were applied to each well. The cells were then harvested and fixed in FixDenat solution for 30 min. BrdU incorporated in cells were then detected by peroxidase-labeled anti BrdU antibodies using TMB as substrate. The results were measured with a microplate reader 3550-UV (Bio-Rad). The relative cell proliferation was calculated as the absorbance at 450 nm (A450) of samples over the A450 of the control cells.

Splenic cells were stained with carboxyfluorescein diacetate succinimidyl ester (CFSE; 0.1 μM; Invitrogen, Carlsbad, CA, USA) for 5 min at 37°C. Excessive CFSE was removed, and the reaction was stopped by applying 1 mL of ice-cold FBS and allowed cell suspension to stand for 5 min. The cells were then suspended in RPMI medium, seeded in 12-well flat-bottom plate at 2.5×10^6^ cell/mL and stimulated with YZP (20 μg/mL) for 72 h. Next, the cells were harvested for flow cytometry analysis. The degree of cell proliferation was determined based on the reduction of CFSE fluorescence.
